# Protein kinase N1 critically regulates cerebellar development and long-term function

**DOI:** 10.1172/JCI96165

**Published:** 2018-04-16

**Authors:** Stephanie zur Nedden, Rafaela Eith, Christoph Schwarzer, Lucia Zanetti, Hartwig Seitter, Friedrich Fresser, Alexandra Koschak, Angus J.M. Cameron, Peter J. Parker, Gottfried Baier, Gabriele Baier-Bitterlich

**Affiliations:** 1 Biocenter, Division of Neurobiochemistry, and; 2 Department of Pharmacology, Medical University of Innsbruck, Innsbruck, Austria.; 3 Institute of Pharmacy, Pharmacology and Toxicology, Center for Molecular Biosciences, University of Innsbruck, Innsbruck, Austria.; 4 Department for Pharmacology and Genetics, Division of Translational Cell Genetics, Medical University of Innsbruck, Innsbruck, Austria.; 5 Kinase Biology Laboratory, John Vane Science Centre, Barts Cancer Institute, Queen Mary University of London, London, United Kingdom.; 6 Francis Crick Institute, London, United Kingdom.; 7 Division of Cancer Studies, King’s College London, London, United Kingdom.

**Keywords:** Neuroscience, Neurodevelopment, Protein kinases, Synapses

## Abstract

Increasing evidence suggests that synapse dysfunctions are a major determinant of several neurodevelopmental and neurodegenerative diseases. Here we identify protein kinase N1 (PKN1) as a novel key player in fine-tuning the balance between axonal outgrowth and presynaptic differentiation in the parallel fiber–forming (PF-forming) cerebellar granule cells (Cgcs). Postnatal *Pkn1*^–/–^** animals showed a defective PF–Purkinje cell (PF-PC) synapse formation. In vitro, *Pkn1^–/–^* Cgcs exhibited deregulated axonal outgrowth, elevated AKT phosphorylation, and higher levels of neuronal differentiation-2 (NeuroD2), a transcription factor preventing presynaptic maturation. Concomitantly, *Pkn1^–/–^* Cgcs had a reduced density of presynaptic sites. By inhibiting AKT with MK-2206 and siRNA-mediated knockdown, we found that AKT hyperactivation is responsible for the elongated axons, higher NeuroD2 levels, and reduced density of presynaptic specifications in *Pkn1^–/–^* Cgcs. In line with our in vitro data, *Pkn1^–/–^* mice showed AKT hyperactivation, elevated NeuroD2 levels, and reduced expression of PF-PC synaptic markers during stages of PF maturation in vivo. The long-term effect of *Pkn1* knockout was further seen in cerebellar atrophy and mild ataxia. In summary, our results demonstrate that PKN1 functions as a developmentally active gatekeeper of AKT activity, thereby fine-tuning axonal outgrowth and presynaptic differentiation of Cgcs and subsequently the correct PF-PC synapse formation.

## Introduction

Protein kinase N1 (PKN1/PRK1) is the most abundantly expressed isoform of the PKN family in the central nervous system and accounts for 0.01% of total brain protein (1). It is widely studied for its involvement in cancer (2); however, surprisingly little is known about the brain-specific function of this kinase, even though it was first isolated from human hippocampal cDNA in 1994 (3) and is particularly enriched in certain brain areas (4). In human neurons PKN is mainly localized to juxtanuclear, cytoplasmic, dendroplasmic, and pre- and postsynaptic compartments (5).

PKN1 is a serine/threonine kinase and belongs to the protein kinase C superfamily, sharing a characteristic C-terminal catalytic domain (3, 6) that requires phosphorylation by PDK-1 for activation. The N-terminal regulatory domain confers binding and regulation by RhoA/B/C, Rac1 (7), fatty acids, and phospholipids (8). Activation of PKN1 is also achieved by caspase-3–mediated cleavage, resulting in a constitutively active protein product missing the regulatory N-terminus (9). This form of deregulated PKN1 activation occurs during apoptosis (10) and has been linked to various insults to the brain (11–14). We have previously reported that PKN1 is part of a purine-nucleoside signaling cascade involved in the protection of hypoxic neuronal cultures and cell lines in vitro (15, 16). However, despite those in vitro reports and evidence on the generation of a constitutively active fragment, the physiological function of PKN1 in the nervous system in vivo is not yet known.

Using *Pkn1^–/–^* animals (17), this work set out to clarify the role of PKN1 in the brain. We focused on the cerebellum, which has a central role in motor control and coordination and is also the brain area with the highest PKN1 expression levels (4). The lengthy process to achieve cerebellar maturity makes it particularly susceptible to developmental abnormalities, which may finally result in neurodegeneration and disabilities such as cerebellar ataxia (18). Two excitatory afferents converge onto Purkinje cells (PCs), the only output neurons of the cerebellum: climbing fibers (CFs) from inferior olivary nuclei, and parallel fibers (PFs) from cerebellar granule cells (Cgcs). A hallmark in cerebellar development is the correct formation of the PF-PC synapse (19), which is important for the segregation of CF and PF territories (20, 21) and cerebellar long-term function (22). PF-PC synaptic dysfunctions have been implicated in models of spinocerebellar ataxias 1, 3, 5, and 27 and Friedreich’s ataxia as well as autism spectrum disorders (19, 23). Considering the high expression levels of PKN1 in Cgcs and PCs (4), we investigated the effect of *Pkn1* deletion on the formation of PF-PC as well as CF-PC synapses during cerebellar development.

Our results demonstrate that during cerebellar development PKN1 functions as a gatekeeper of AKT activity and subsequently protein levels of the transcription factor neuronal differentiation-2 (NeuroD2), thereby fine-tuning axonal outgrowth and presynaptic differentiation of Cgcs. Accordingly, *Pkn1* deletion results in disrupted PF-PC synapse formation and defective CF elimination, as seen in a reduced expression of the PF-PC synaptic marker cerebellin 1 (Cbln1), persistent multiple CF innervation, and reduced spontaneous PC activity. The long-term effect of *Pkn1* deletion was further seen in cerebellar atrophy and mild ataxia in adult *Pkn1^–/–^* animals. Despite the rapidly increasing literature on AKT signaling and neurodevelopment, this is, to our knowledge, the first report linking developmental AKT activity with NeuroD2 levels and cerebellar synapse formation, and we identify PKN1 as a regulator of this pathway.

## Results

### Deletion of Pkn1 leads to a defective PF-PC synapse formation and PC activity.

**Figure 1:**
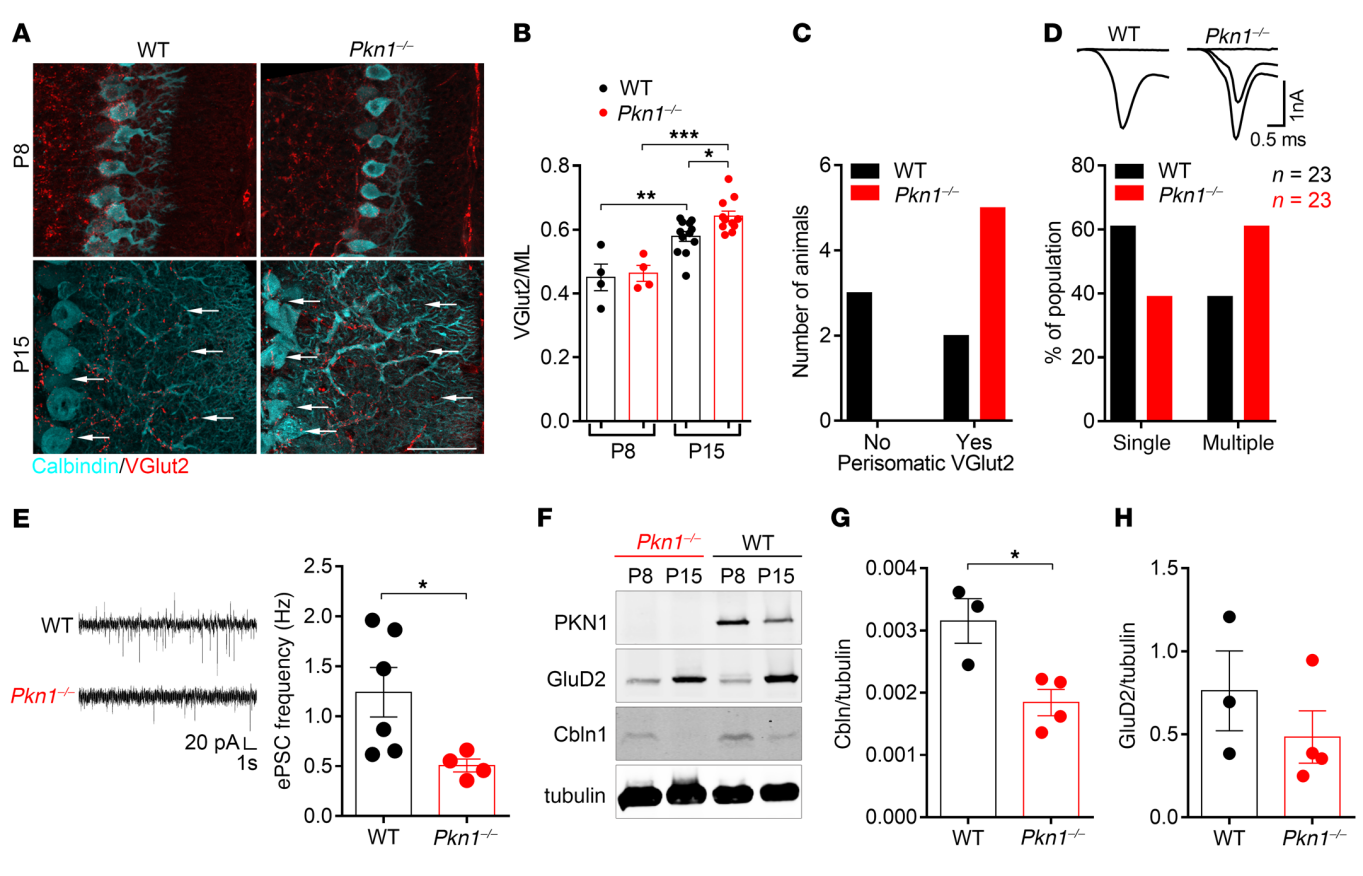
*Pkn1^–/–^* mice show a defective PF-PC synapse formation during development. (**A**) Cerebellar vermis sections of P8–P15 animals (*n* = 4–12). Arrows mark distal and perisomatic varicosities of VGlut2-stained CFs. Scale bar: 50 μm. (**B**) The ratio of the VGlut2-stained CF innervation depth (μm) to the ML thickness (μm) was analyzed [1-way ANOVA with Newman-Keuls multiple-comparisons test, *F*(3,27) = 16.7, *P* < 0.0001, post-test *P < 0.05, **P < 0.01, ***P < 0.001; *n* = 4 WT, 4 *Pkn1^–/–^* animals for P8, *n* = 12 WT, 11 *Pkn1^–/–^* animals for P15 from 5–8 litters per group]. (**C**) The score of PC perisomatic VGlut2 staining in P15 animals [χ^2^ test = 4.286, *P* = 0.0384, *n* = 5 WT, 5 *Pkn1^–/–^* animals from 5 litters per group]. (**D**) CF-induced ePSCs were recorded from PCs in acute slices. With increasing stimulation strength, ePSCs were obtained in an all-or-none fashion (single CF) or in 2 or more discrete steps (multiple CFs) [χ^2^ test = 9.68, *P* = 0.0019, *n* = 23 WT, 23 *Pkn1^–/–^* cells from 7 P15–P17 animals per group]. (**E**) Spontaneous PC ePSC frequencies [2-tailed unpaired *t* test with Welch’s correction, *t*(5) = 2.865, *P = 0.0352, *n* = 6 WT, 4 *Pkn1^–/–^* cells from 3–5 P13–P15 animals per group]. (**F**) Western blot analysis of Cbln1 and GluD2 levels (*n* = 3–4). (**G** and **H**) Analysis of the Cbln1/tubulin ratio (**G**) [2-tailed unpaired *t* test, *t*(5) = 3.365, *P = 0.0200, *n* = 3 WT, 4 *Pkn1^–/–^* extracts from 3–4 animals per group] and GluD2/tubulin ratio (**H**) [2-tailed unpaired *t* test, *t*(5) = 1.016, *P* = 0.3561, *n* = 3 WT, 4 *Pkn1^–/–^* extracts from 3–4 animals per group] in P15 animals. Data are presented as individual *n* values with mean ± SEM. All analyses/experiments except **F**–**H** were performed in a blinded manner.

We first analyzed CF growth, as an indicator of a functioning PF-PC synapse formation, by staining of cerebellar sections of postnatal day 8 (P8)–P15 WT and *Pkn1^–/–^* animals with the CF-specific marker vesicular glutamate transporter 2 (VGlut2) (20). Early during cerebellar development, PC somata are innervated by multiple CFs. From P9 onward a single “winner” CF starts dendritic translocation and expands its territory (20). Perisomatic CF synapse elimination occurs in an early, PF-independent phase (~P7–P11) and a late phase (~P12–P17), which, similar to the proximal dendritic restriction of CF innervation, strictly depends on a functioning PF-PC synapse (21). There were no differences between WT and *Pkn1^–/–^* animals in VGlut2-stained CF terminals at P8, where they were mainly found around the PC somata (Figure 1, A and B). However, as compared with WT animals, cerebella of P15 *Pkn1^–/–^* mice showed an enhanced distal extension of CF terminals into PF territory (Figure 1, A and B) and a defective perisomatic CF elimination (Figure 1, A and C). Western blot analysis further revealed that the ratio of VGlut2 to the PF-specific marker vesicular glutamate transporter 1 (VGlut1) (20) dropped from P8 to P15 in WT animals but stayed the same in *Pkn1^–/–^* animals (Supplemental Figure 1A; supplemental material available online with this article; https://doi.org/10.1172/JCI96165DS1), further showing imbalances in CF/PF innervation. VGlut1 expression was consistently lower in *Pkn1^–/–^* animals during development (Supplemental Figure 1A). Starting at P15, we detected dendritic thickening of *Pkn1^–/–^* PCs that coincided with the defective CF growth (Supplemental Figure 1B). At these early developmental stages, those defects did not translate into altered cerebellar morphology of *Pkn1^–/–^* mice. WT and *Pkn1^–/–^* mice showed a similar cerebellar size, foliation pattern, and thickness of the external granule layer (EGL), internal granule layer (IGL), and molecular layer (ML) (Supplemental Figure 1C).

To reveal potential CF synapse elimination deficits in *Pkn1^–/–^* animals, we measured CF-induced excitatory postsynaptic currents (ePSCs) in PCs in acute slices prepared from P15–P17 animals (24). With gradually increasing stimulus intensities, the majority of ePSCs of WT PCs were obtained in an all-or-none fashion, while the majority of ePSCs of *Pkn1^–/–^* PCs occurred at 2 or more discrete steps (Figure 1D). This indicates a more frequent occurrence of multiple CF innervation in *Pkn1^–/–^* mice.

To further expose a functional defect in PF-PC synapse formation, we recorded spontaneous ePSCs of PCs in acute slices prepared from P13–P15 WT and *Pkn1^–/–^* animals. Recordings were performed at room temperature to avoid intrinsic PC firing (25), and therefore ePSCs mainly reflect PF synapse activity (26, 27). Interestingly, *Pkn1^–/–^* PCs showed reduced ePSC frequencies (Figure 1E) but similar ePSC amplitudes (Supplemental Figure 1D), indicating differences in the number of functional synapses but not in presynaptic quantal content or postsynaptic receptors. Likewise, *Pkn1^–/–^* cerebellar slices had a reduced inhibitory PSC (iPSC) input (Supplemental Figure 1E), which might also be caused by a defective PF-ML interneuron synapse formation.

We next tested the expression of the PF-PC synaptic markers cerebellin 1 (Cbln1), a protein excreted by Cgcs and important for PF-PC synapse stabilization (22, 28), and δ2 glutamate receptor (GluD2), the PC postsynaptic receptor binding to extracellular Cbln1 (29). Consistently with a defective PF-PC synapse formation, Cbln1 expression levels were lower in P15 *Pkn1^–/–^* cerebella (Figure 1, F and G). GluD2 expression levels were, however, only marginally affected (Figure 1, F and H), suggesting a Cgc-specific defect. We next screened in vitro Cgcs for differences in presynaptic maturation and axonal outgrowth properties, since the correct balance between axonal growth and presynaptic differentiation is an essential part of synapse formation (30).

### PKN1 regulates axonal outgrowth and the density of presynaptic sites in Cgcs in vitro.

**Figure 2:**
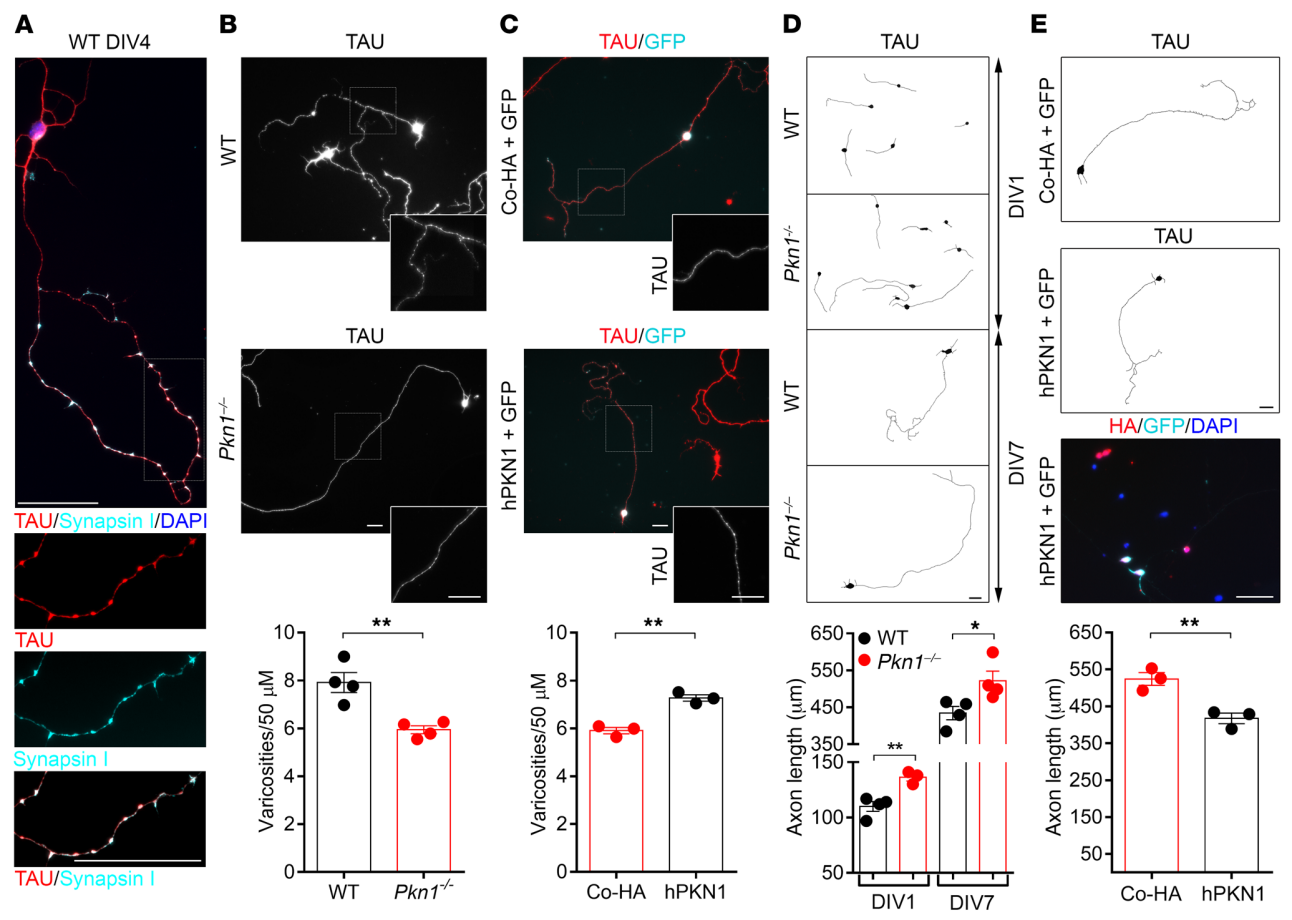
*Pkn1^–/–^* Cgcs have a reduced density of presynaptic sites and enhanced axonal outgrowth. (**A**) In WT Cgcs, TAU-stained axonal *en passant* swellings colocalized with the presynaptic marker synapsin I (images were taken at DIV4 and are representative of at least 3 separate experiments in WT and *Pkn1^–/–^* Cgcs). (**B**) The number of *en passant* swellings (varicosities) per axonal section was analyzed in WT and *Pkn1^–/–^* Cgcs at DIV7 [2-tailed unpaired *t* test, *t*(6) = 4.413, **P = 0.0045, *n* = 4 WT, 4 *Pkn1^–/–^* Cgc preparations from 4 litters per group]. (**C**) *Pkn1^–/–^* Cgcs were transfected with *GFP* together with a control HA-plasmid (Co-HA) or human HA-tagged *PKN1* (h*PKN1*), and varicosities per axonal section were analyzed at DIV7 in GFP-expressing/TAU-stained Cgcs [2-tailed unpaired *t* test, *t*(4) = 7.147, **P = 0.002, *n* = 3 from 3 litters]. (**D**) Axonal length after DIV1 [2-tailed unpaired *t* test, *t*(5) = 4.431, **P = 0.0068, *n* = 4 WT, 3 *Pkn1^–/–^* Cgc preparations from 3–4 litters per group] and DIV7 [2-tailed unpaired *t* test, *t*(6) = 2.692, *P = 0.0360, *n* = 4 WT, 4 *Pkn1^–/–^* Cgc preparations from 4 litters per group] in TAU-stained Cgcs. (**E**) *Pkn1^–/–^* Cgcs were transfected with *GFP* together with Co-HA or h*PKN1*, and axonal length at DIV7 was analyzed in GFP-expressing/TAU-stained Cgcs [2-tailed unpaired *t* test, *t*(4) = 4.752, **P = 0.0090, *n* = 3 from 3 litters]. GFP-expressing cells also expressed hPKN1, as seen in overlapping GFP/HA staining (image is representative of 3 separate experiments). Data are presented as individual *n* values with mean ± SEM. Cgcs were grown on laminin-coated coverslips, and representative WIS-NeuroMath–analyzed output images are shown in **D** and **E**. All scale bars: 50 μm. Experimenters were not blinded to the genotype or treatment.

We first analyzed mature Cgc cultures (4–7 days in vitro [DIV]) for differences in presynaptic sites, which appear as “*en passant* swellings” (31) along the axon. These varicosities show colocalization of TAU and the presynaptic marker synapsin I (Figure 2A). In *Pkn1^–/–^* Cgcs transfected with HA-tagged human *PKN1* (h*PKN1*), HA staining was found around the nucleus, in dendrites, and along those *en passant* swellings of the axon (Supplemental Figure 2, A and B). Interestingly, mature *Pkn1^–/–^* Cgc cultures had a reduced density of presynaptic sites (Figure 2B), an effect that could be rescued by reintroduction of h*PKN1* (Figure 2C). *Pkn1* knockout also resulted in deregulated axonal outgrowth, as seen in elongated axons of *Pkn1^–/–^* Cgcs throughout the entire culture period (Figure 2D). The enhanced axonal outgrowth was reduced to WT levels in *Pkn1^–/–^* Cgcs transfected with h*PKN1* (Figure 2E). These results point toward elongated axonal outgrowth at the expense of presynaptic differentiation in *Pkn1^–/–^* Cgcs. We therefore next screened Cgc protein extracts for differences in PKN1 downstream signaling molecules involved in presynaptic differentiation and axonal outgrowth.

### Pkn1 knockout results in enhanced AKT phosphorylation and NeuroD2 expression in Cgcs in vitro.

**Figure 3:**
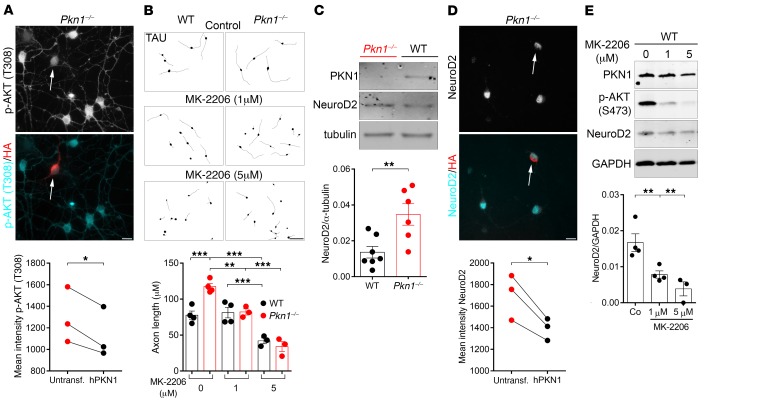
*Pkn1* knockout results in elevated AKT phosphorylation and NeuroD2 protein levels in Cgcs in vitro. (**A**) Phospho-AKT (p-AKT) [T308] intensity was measured in untransfected *Pkn1^–/–^* Cgcs and *Pkn1^–/–^* Cgcs expressing human HA-tagged PKN1 (hPKN1) [8–22 transfected cells were analyzed per experiment; 2-tailed paired *t* test, *t*(2) = 5.365, *P = 0.033, *n* = 3 from 3 litters]. (**B**) Cgc axonal length was measured at DIV1 after 24 hours of treatment with the AKT inhibitor MK-2206 (1 or 5 μM) in TAU-stained Cgcs [1-way ANOVA with Newman-Keuls multiple-comparisons test, *F*(5,15) = 26.97, *P* < 0.0001, post-test **P < 0.01, ***P < 0.001; *n* = 3–4 WT, 3–4 *Pkn1^–/–^* Cgc preparations from 3–4 litters per group]. (**C**) NeuroD2 expression levels were analyzed in Cgcs at DIV6–8 [2-tailed unpaired *t* test, *t*(11) = 3.228, **P = 0.008, *n* = 7 WT, 6 *Pkn1^–/–^* Cgc preparations from 6–7 different litters]. Representative WIS-NeuroMath–analyzed output images are shown. (**D**) NeuroD2 intensity was measured at DIV4 in untransfected *Pkn1^–/–^* Cgcs and *Pkn1^–/–^* Cgcs expressing hPKN1 [26–32 transfected cells were analyzed per experiment; 2-tailed paired *t* test, *t*(2) = 4.904, *P = 0.0392, *n* = 3 from 3 litters]. (**E**) WT Cgc protein extracts at DIV1 were analyzed for the effect of 24 hours of treatment with MK-2206 on NeuroD2 protein levels [1-way ANOVA with Newman-Keuls multiple-comparisons test, *F*(2,8) = 11.76, *P* = 0.0042, post-test **P < 0.01, *n* = 3–4 from 3–4 litters]. All data are presented as individual *n* values with mean ± SEM. Scale bars: 10 μm in **A** and **D**, 50 μm in **B**. Experimenters were not blinded to the genotype or treatment.

An important regulator of axonal outgrowth is the protein kinase AKT (32), and PKN1 has been previously suggested to negatively regulate AKT activity (33), for example downstream of the B cell antigen receptor (34). We found that Cgcs from *Pkn1^–/–^* mice showed significantly higher endogenous AKT phosphorylation levels at T308 and S473 (Supplemental Figure 2C). The mean phospho-AKT (p-AKT) T308 (Figure 3A) and p-AKT S473 (Supplemental Figure 2D) intensity of *Pkn1^–/–^* Cgcs was consistently reduced in h*PKN1*-transfected cells, but not in *GFP*-transfected cells (Supplemental Figure 2E), showing that the higher AKT phosphorylation was specifically caused by the absence of PKN1. We next tested whether *Pkn1* knockout–mediated AKT hyperactivation is the cause of elongated axonal outgrowth, by incubating Cgcs with the potent AKT inhibitor MK-2206 (Supplemental Figure 3A). MK-2206 reduced the axonal length of *Pkn1^–/–^* Cgcs to WT levels (Figure 3B), establishing PKN1 as a regulator of axonal length upstream of AKT.

Interestingly, we found that mature *Pkn1^–/–^* Cgc cultures had higher NeuroD2 protein levels (Figure 3C). Transfection of *Pkn1^–/–^* Cgcs with h*PKN1*, but not with *GFP* (Supplemental Figure 3B), reduced the mean NeuroD2 intensity in immunofluorescence stainings (Figure 3D), establishing PKN1 as a negative regulator of NeuroD2 levels. This fits well with our observation of a defective spacing of presynaptic sites in *Pkn1^–/–^* Cgcs (Figure 2B), since NeuroD2 is a transcription factor preventing presynaptic differentiation, whose overexpression reduces the density of presynaptic sites in Cgcs (30). Since AKT has been shown to enhance the activity of several transcription factors regulating NeuroD2 expression, such as neurogenin 1 and neuronal differentiation-1 (NeuroD1) (35, 36), we next tested whether AKT regulates NeuroD2 protein levels in Cgcs.

In protein extracts of WT Cgcs at DIV1 treated with MK-2206 for 24 hours, we found that MK-2206 dose-dependently reduced NeuroD2 levels (Figure 3E). Additionally, we detected enhanced Cbln1 expression upon inhibition of AKT (Supplemental Figure 3C). MK-2206 had similar effects in *Pkn1^–/–^* Cgcs (Supplemental Figure 3D). Furthermore, *Pkn1^–/–^* Cgcs showed a trend toward reduced Cbln1 levels (Supplemental Figure 3E). To further validate those results, we next suppressed AKT expression in order to see whether the observed phenotype of *Pkn1^–/–^* Cgcs could be restored to WT levels.

**Figure 4:**
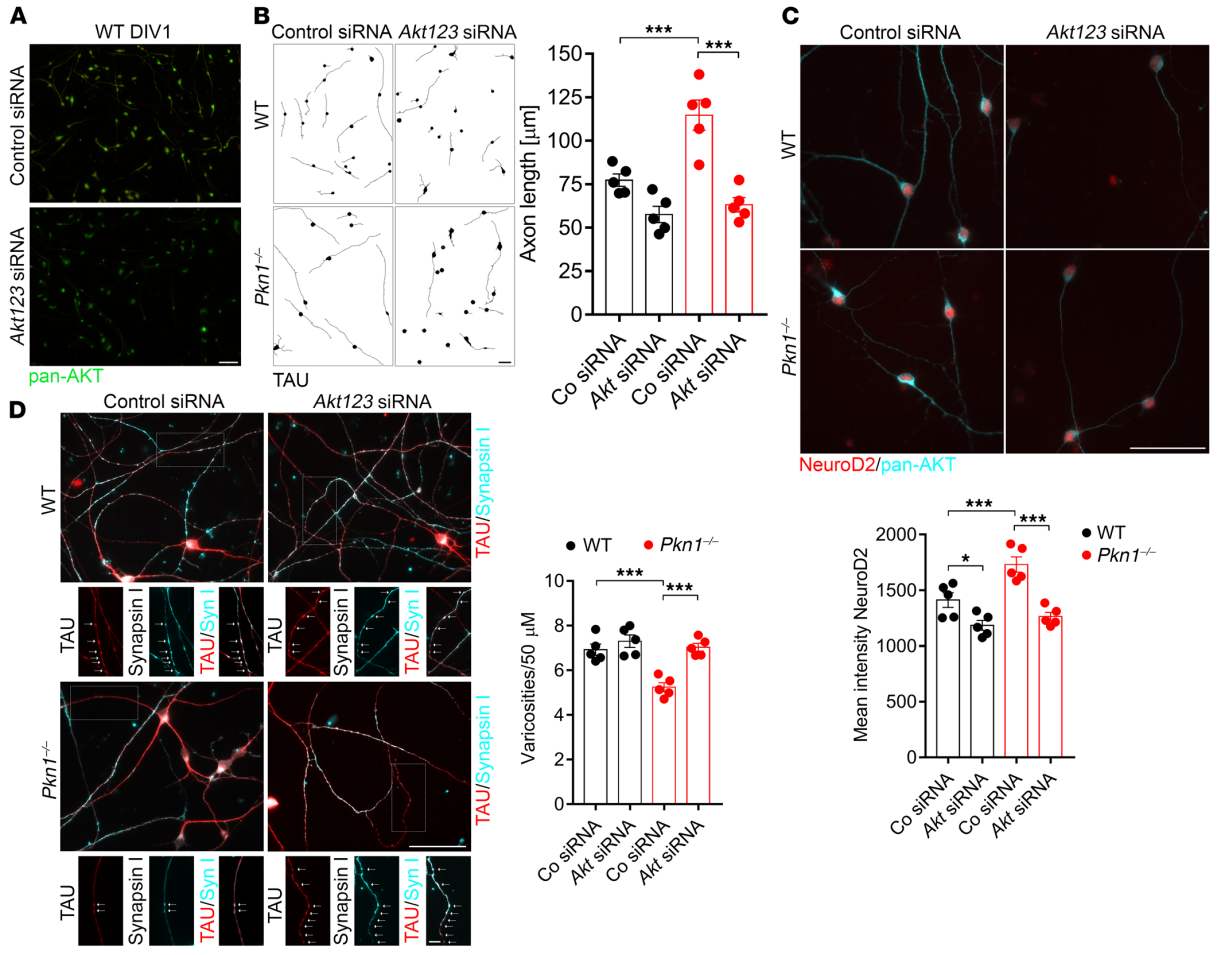
siRNA-mediated *Akt* knockdown reduces axonal length and NeuroD2 protein levels and increases the density of presynaptic sites in *Pkn1^–/–^* Cgcs. (**A**) siRNAs targeting *Akt123* reduce pan-AKT expression after DIV1. Pictures are representative of 5 separate experiments. For analysis at DIV1 and DIV4 in WT and *Pkn1^–/–^* Cgcs, see Supplemental Figure 4, A and B. (**B**) siRNAs targeting *Akt123* significantly reduce axonal length of *Pkn1^–/–^* Cgcs at DIV1 [1-way ANOVA with Newman-Keuls multiple-comparisons test, *F*(3,16) = 20.78, *P* < 0.0001, post-test ***P < 0.001, *n* = 5 WT, 5 *Pkn1^–/–^* Cgc preparations from 3–5 litters per group]. Axons were stained with TAU. Representative WIS-NeuroMath–analyzed output images are shown. (**C**) siRNAs targeting *Akt123* significantly reduce NeuroD2 intensity in WT and *Pkn1^–/–^* Cgcs at DIV4 [1-way ANOVA with Newman-Keuls multiple-comparisons test, *F*(3,16) = 18.73, *P* < 0.0001, post-test *P < 0.05, ***P < 0.001; *n* = 5 WT, 5 *Pkn1^–/–^* Cgc preparations from 3–5 litters per group]. (**D**) Presynaptic sites (varicosities) were stained with TAU and synapsin I. Insets represent higher-magnification single- and double-labeled examples of axonal varicosities (indicated by arrows). White varicosities in double-labeled insets demonstrate TAU and synapsin I colocalization. siRNAs targeting *Akt123* significantly increase the density of presynaptic sites in *Pkn1^–/–^* Cgcs at DIV4 [1-way ANOVA with Newman-Keuls multiple-comparisons test, *F*(3,16) = 16.62, *P* < 0.0001, post-test ***P < 0.001, *n* = 5 WT, 5 *Pkn1^–/–^* Cgc preparations from 3–5 litters per group]. All data are presented as individual *n* values with mean ± SEM. Scale bars: 50 μm, except in inset in **D**: 10 μm. Experimenters were not blinded to the genotype or treatment.

*siRNA-mediated knockdown of Akt123 restores axonal length, NeuroD2 expression levels, and the density of presynaptic sites in Pkn1^–/–^Cgcs to WT levels*. WT and *Pkn1^–/–^* Cgcs were transfected with siRNAs targeting *Akt123* or control nontargeting siRNAs and stained for pan-AKT. *Akt123* siRNAs significantly reduced pan-AKT expression at DIV1 and DIV4 (Figure 4A and Supplemental Figure 4, A and B). The concentration of *Akt* siRNAs was chosen to accomplish a significant decrease in AKT without adverse effect on cell viability. Knockdown of *Akt123* significantly reduced the enhanced axonal length of *Pkn1^–/–^* Cgcs at DIV1 (Figure 4B). Similarly, elevated NeuroD2 levels in *Pkn1^–/–^* Cgcs were restored to WT levels upon *Akt123* knockdown (Figure 4C) and accompanied by an increased density of presynaptic sites (Figure 4D). Knockdown of *Akt123* also resulted in an enhanced expression of Cbln1 in WT (Supplemental Figure 4C) and *Pkn1^–/–^* Cgcs (Supplemental Figure 4D). These data confirm our previous findings that PKN1-mediated modulation of AKT is crucial for the balance between axonal outgrowth, NeuroD2/Cbln1 expression, and presynaptic differentiation. We therefore next tested AKT phosphorylation and NeuroD2 expression during in vivo development in WT and *Pkn1^–/–^* animals.

### Cerebellar alterations in Pkn1^–/–^ mice coincide with developmentally enhanced AKT phosphorylation and NeuroD2 expression in vivo.

**Figure 5:**
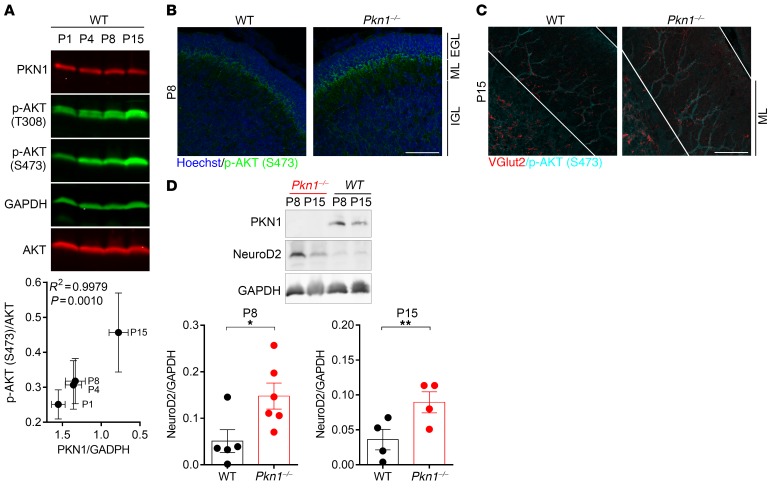
*Pkn1^–/–^* cerebella show elevated AKT phosphorylation and NeuroD2 protein levels during developmental stages of PF maturation. (**A**) There was a significant correlation between the p-AKT [S473]/AKT ratio and the PKN1/GAPDH ratio in cerebellar protein extracts from P1–P15 WT animals (Pearson correlation, number of XY pairs: 4, Pearson *r* = –0.9990, 2-tailed, *P* = 0.001, *R*^2^ = 0.9979, *n* = 3–4 from 3–4 litters per group). Data are presented as mean ± SEM. (**B** and **C**) Confocal images of cerebellar sections of P8 and P15 WT and *Pkn1^–/–^* animals stained for p-AKT [S473] and Hoechst (P8) (**B**) or p-AKT [S473] and VGlut2 (P15) (**C**). Pictures are representative of at least 3 independent experiments. (**D**) Western blot analysis of NeuroD2 expression in P8 [2-tailed unpaired *t* test, *t*(9) = 2.546, *P = 0.0314, *n* = 5 WT, 6 *Pkn1^–/–^* animals from 4–5 different litters] and in P15 WT and *Pkn1^–/–^* whole cerebella protein extracts [2-tailed unpaired *t* test, *t*(6) = 2.541, **P = 0.0440, *n* = 4 WT, 4 *Pkn1^–/–^* animals from 4–5 different litters]. Data are presented as individual *n* values with mean ± SEM. All scale bars: 50 μm. Experimenters were not blinded to the genotype.

Protein lysates prepared from P1–P15 WT cerebella showed an inverse correlation between PKN1 expression dropping and AKT phosphorylation increasing during development (Figure 5A). Concomitantly we found higher AKT phosphorylation in *Pkn1^–/–^* cerebella protein lysates (Supplemental Figure 5A) and in immunofluorescence stainings (Figure 5, B and C). AKT phosphorylation levels were particularly increased in areas and developmental stages of axonal outgrowth and maturation of Cgcs and dendritic outgrowth and maturation of PCs. At P8 *Pkn1^–/–^* animals showed higher AKT phosphorylation in the PF-forming Cgcs of the premigratory EGL, where Cgcs start extending axons, as well as in the IGL (Figure 5B). At P15, higher AKT phosphorylation was found in the IGL and in PC dendrites (Figure 5C). In agreement with greater AKT activity, we found increased NeuroD2 protein levels in *Pkn1^–/–^* cerebellar protein extracts (Figure 5D). There were no differences in AKT phosphorylation or NeuroD2 levels in adult animals (Supplemental Figure 5, B and C), showing a development-specific effect of *Pkn1* knockout on AKT and NeuroD2.

These exciting results show, for the first time to our knowledge, that PKN1 controls AKT phosphorylation and NeuroD2 expression during cerebellar development in vivo, thereby explaining the defective PF-PC synapse formation and reduced Cbln1 expression levels upon *Pkn1* knockout.

Interestingly, we also found that the enlarged dendritic caliber of *Pkn1^–/–^* PCs could be reduced to WT levels upon incubation of organotypic slices with the AKT antagonist MK-2206, showing that PKN1 also controls PC dendritic caliber upstream of AKT (Supplemental Figure 5D). Therefore, we cannot exclude a PC-dependent defect, due to dendritic thickening upon *Pkn1* knockout, that further weakens PF-PC synapse formation.

**Figure 6:**
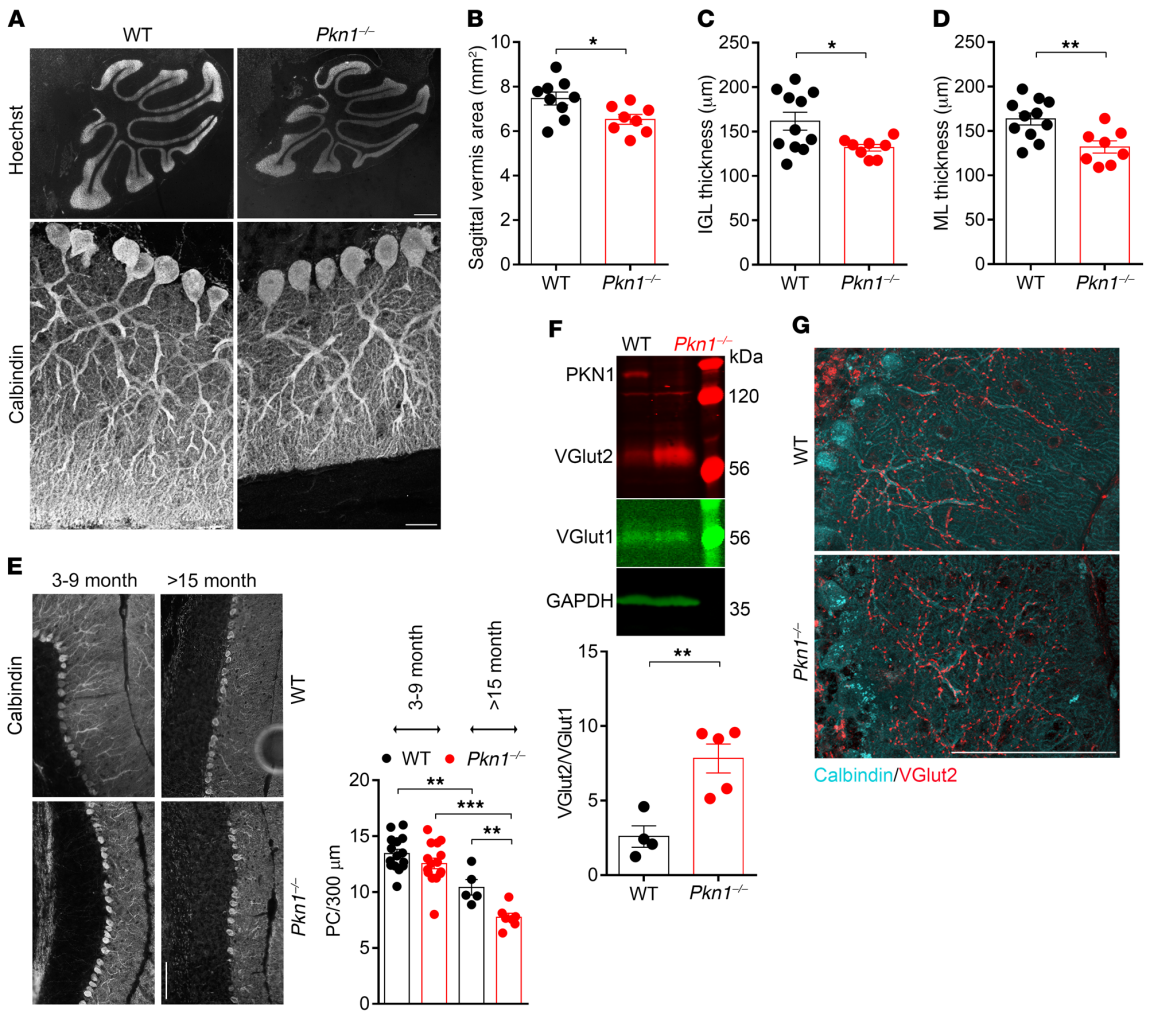
Adult *Pkn1^–/–^* mice show cerebellar shrinkage and late-onset PC degeneration. (**A**) Size differences of adult WT and *Pkn1^–/–^* cerebella (3–9 months), as seen in Hoechst-stained sagittal vermis sections and calbindin-stained ML pictures (representative images of 8–11 separate experiments). (**B**–**D**) Analysis of the cerebellar vermis area (**B**) [2-tailed unpaired *t* test, *t*(15) = 2.510, *P = 0.0236, *n* = 9 WT, 8 *Pkn1^–/–^* animals from 4–6 litters per group], the IGL thickness (**C**) [2-tailed unpaired *t* test with Welch’s correction, *t*(12) = 2.772, *P = 0.0169, *n* = 11 WT, 8 *Pkn1^–/–^* animals from 4–5 litters per group], and the ML thickness (**D**) [2-tailed unpaired *t* test, *t*(17) = 3.210, **P = 0.0051, *n* = 11 WT, 8 *Pkn1^–/–^* animals from 4–5 litters per group] in 3- to 9-month-old animals. (**E**) PC number in 3- to 9-month-old and 15- to 22-month-old animals [1-way ANOVA with Newman-Keuls multiple-comparisons test, *F*(3,38) = 23.12, *P* < 0.0001, post-test **P < 0.01, ***P < 0.001; *n* = 5–15 WT, 7–15 *Pkn1^–/–^* animals]. (**F**) Cerebellar protein extracts from 3- to 9-month-old animals were analyzed for the VGlut2/VGlut1 ratio [2-tailed unpaired *t* test, *t*(7) = 4.138, **P = 0.0044, *n* = 4 WT, 5 *Pkn1^–/–^* animals from 4 litters per group]. (**G**) Calbindin- and VGlut2-stained cerebellar sections of 3- to 9-month-old animals (representative images of 11–12 experiments; see Supplemental Figure 6C for analysis). All data are presented as individual *n* values with mean ± SEM. Scale bars: **A**, Hoechst, 500 μm; calbindin, 50 μm; **E** and **G**, 100 μm. All analyses were performed by experimenters blinded to the genotype, except **F**.

*Adult Pkn1^–/–^animals show cerebellar degeneration*. Several other studies have related a defective PF-PC synapse formation to a degeneration of Cgcs and a late-onset loss of PCs (22, 37). As compared with WT mice, adult (3–9 months old) *Pkn1^–/–^* mice still displayed a similar cerebellar foliation pattern, but *Pkn1^–/–^* animals had smaller cerebella, as seen in a smaller sagittal vermis areas (Figure 6, A and B), with a thinner IGL (Figure 6, A and C) and ML (Figure 6, A and D). The facts that the proliferative layer of the EGL in young animals was similar between both genotypes (Supplemental Figure 6A) and that there were no ectopic NeuN-positive cells in the ML of adult *Pkn1^–/–^* cerebella (Supplemental Figure 6B) rule out a defective proliferation/migration of *Pkn1^–/–^* Cgcs as the underlying mechanism. In further agreement with a defective PF-PC synapse formation, we saw no significant PC degeneration in 3- to 9-month-old animals, but we found a late-onset loss of PCs in *Pkn1^–/–^* mice older than 15 months (Figure 6E).

Adult *Pkn1^–/–^* mice still showed abnormal CF innervation, as seen in a significantly higher ratio of VGlut2/VGlut1 protein levels (Figure 6F) and increased VGlut2 staining (Figure 6G). In WT animals, VGlut2-stained CF terminals showed a reduction in the number of varicosities from the proximal part of the PC dendrite to the distal part (Figure 6G and Supplemental Figure 6C). This was not seen in *Pkn1^–/–^* animals, where the number of varicosities remained the same throughout the entire innervation depth of CFs (Figure 6G and Supplemental Figure 6C). Additionally, PCs of adult *Pkn1^–/–^* animals still had thicker dendrites (Supplemental Figure 6D), showing that *Pkn1* knockout–mediated defects of CF elimination and dendritic outgrowth persist throughout life.

**Figure 7:**
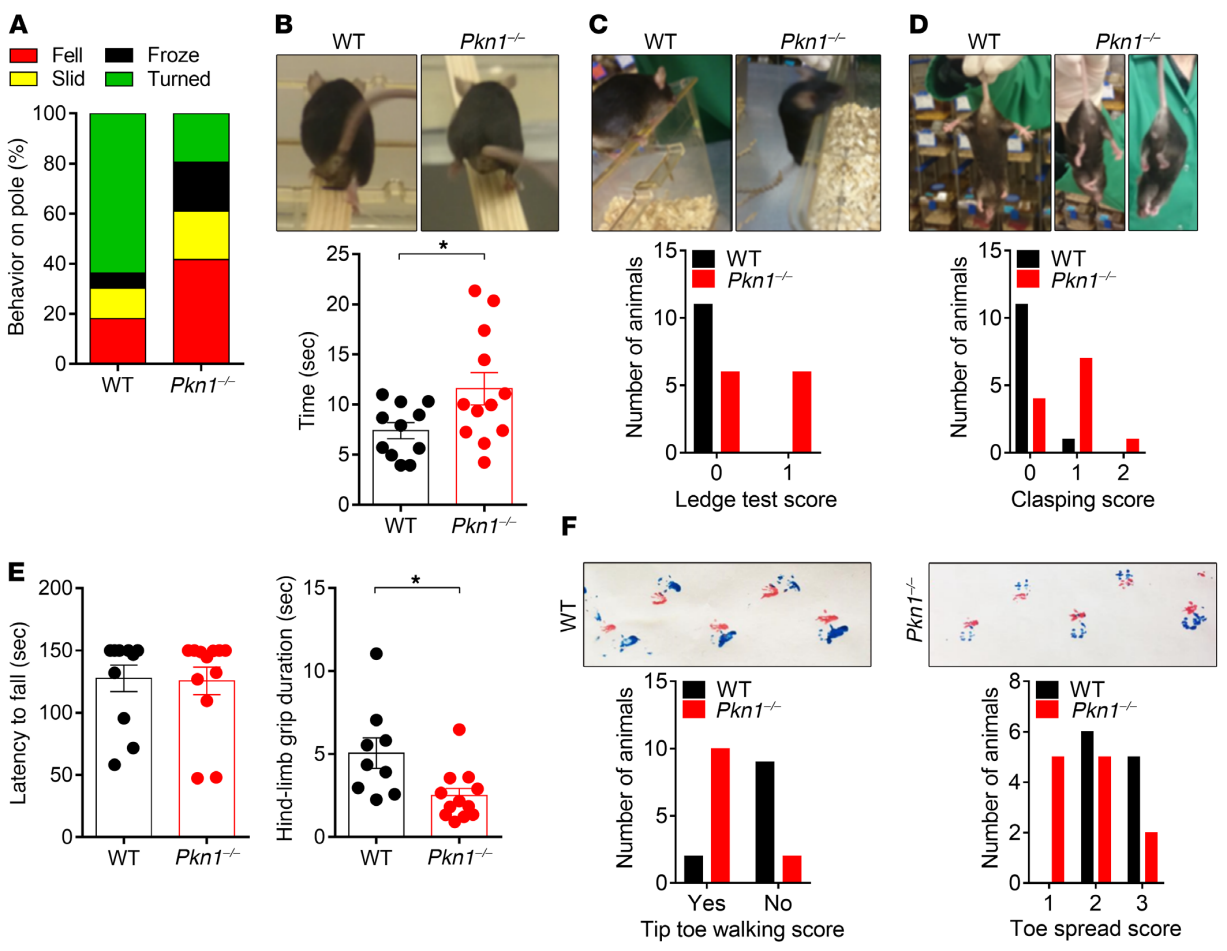
Adult *Pkn1^–/–^* animals have problems with balance and motor coordination. A cohort of adult (4–9 months) WT and *Pkn1^–/–^* animals was examined for motor deficits in a set of behavioral tests. (**A**) Adult mice were tested in the vertical pole task [χ^2^ test (3) = 42.33, *P* < 0.0001, *n* = 11 WT, 12 *Pkn1^–/–^* animals from 4–6 litters per group]. Data are presented as mean percentage. (**B**) Animals were tested in the balance beam test [2-tailed unpaired *t* test with Welch’s correction, *t*(15) = 2.328, *P = 0.0334, *n* = 11 WT, 12 *Pkn1^–/–^* animals from 4–6 litters per group]. (**C**) Animals were scored in the ledge test [χ^2^ test (1) = 7.441, *P* = 0.0064, *n* = 11 WT, 12 *Pkn1^–/–^* animals from 4–6 litters per group]. (**D**) Animals were scored for hind-limb clasping [χ^2^ test (2) = 8.767, *P* = 0.0125, *n* = 11 WT, 12 *Pkn1^–/–^* animals from 4–6 litters per group]. (**E**) In the wire hang test, the grip strength, as determined by the latency to fall [2-tailed unpaired *t* test, *t*(21) = 0.1352, *P* = 0.8938, *n* = 11 WT, 12 *Pkn1^–/–^* animals from 4–6 litters per group] and hind-limb grip duration [2-tailed unpaired *t* test, *t*(19) = 2.724, *P = 0.0135, *n* = 9 WT, 12 *Pkn1^–/–^* animals from 4–6 litters per group], was assessed. (**F**) Footprints were scored for tip toe walking [χ^2^ test (1) = 9.763, *P* = 0.0018, *n* = 11 WT, 12 *Pkn1^–/–^* animals from 4–6 litters per group] and toe spread [χ^2^ test (2) = 6.345, *P* = 0.0419, *n* = 11 WT, 12 *Pkn1^–/–^* animals from 4–6 litters per group]. Data in **B** and **E** are presented as individual *n* values with mean ± SEM. Experimenters were not blinded to the genotype, except in **F**.

*Behavioral phenotyping of adult Pkn1^–/–^mice reveals an ataxia-like phenotype*. Considering the important role of the cerebellum in balance and motor control, we tested a cohort of adult (4–9 months) WT and *Pkn1^–/–^* mice in a set of refined motor behavior tests. *Pkn1^–/–^* mice showed an abnormal performance in the vertical pole test. While the majority of WT mice turned around and climbed down, most *Pkn1^–/–^* mice fell down, slid sideways, or froze on the pole (Figure 7A), indicating balance and motor coordination problems. In line with this, *Pkn1^–/–^* mice were slower than WT mice in crossing a horizontal beam (Figure 7B) and showed more slips and balance coordination problems than WT mice in the ledge test (Figure 7C). Moreover, *Pkn1^–/–^* mice exhibited hind-limb clasping, a sign of neurodegeneration (38), with most animals having one hind limb partly retracted toward the body (Figure 7D). While the grip strength in the wire hang test was not different between the groups, the hind-limb grip duration was significantly reduced in *Pkn1^–/–^* mice, with most mice turning in circles and not being able to grab the wire properly (Figure 7E). Footprint analysis further indicated that *Pkn1^–/–^* mice preferred tip toe walking and showed a reduced toe spread score (Figure 6F). General locomotion in the open field test was similar between WT and *Pkn1^–/–^* mice (Supplemental Figure 7A). Likewise, anxiety-related behavior tested in the elevated plus maze was not affected by *Pkn1* knockout (Supplemental Figure 7B). Therefore, these behavioral tests revealed that *Pkn1^–/–^* mice show normal locomotor activity but have problems with balance and motor coordination and display signs of mild ataxia, such as hind-limb clasping and gait abnormalities. Interestingly, the behavioral abnormalities of *Pkn1^–/–^* mice start before an obvious PC loss (Figure 6E), suggesting synaptic dysfunctions and Cgc degeneration rather than PC degeneration as the underlying mechanism.

## Discussion

Data presented here shed light on the largely unknown brain-specific functions of PKN1. We demonstrate that PKN1 is an important gatekeeper of intrinsic AKT activity during cerebellar development in vivo. We propose a mechanism by which PKN1-mediated AKT inhibition during PF growth (P4–P15) results in a reduction of NeuroD2 levels and a subsequent increase in presynaptic specifications and Cbln1 expression in Cgcs, which is essential for a correct PF-PC synapse formation and cerebellar long-term function.

Accordingly, *Pkn1^–/–^* animals have an impaired developmental regression of CFs, persistent multiple CF innervation, and a reduced spontaneous ePSC frequency of PCs, all indicative of a defective PF-PC synapse formation (Figure 1). Spontaneous PC activity in vitro is highly temperature sensitive and is inhibited at room temperature (25); therefore ePSCs recorded in our setting most likely arise from extrinsic input. Since the ratio of PF to CF synapses in the PC is on the order of 150:1 (39), it is generally assumed that most spontaneous ePSCs reflect PF activity (26, 27). Defective CF elimination and reduced spontaneous PC activity are also seen in animals lacking the *Cbln1* or *GluD2* gene, both of which are needed for a correct PF-PC synapse formation (22, 28, 37). Interestingly, we found a reduced Cbln1 expression in *Pkn1^–/–^* animals, while GluD2 levels were only marginally affected, pointing toward a presynaptic Cgc-specific defect in PF-PC synapse formation.

Using in vitro Cgc cultures, we could show that *Pkn1* knockout leads to enhanced AKT phosphorylation and subsequently higher NeuroD2 protein levels. Cbln1 and NeuroD2 levels are reciprocally regulated by AKT in vitro, with decreased NeuroD2 and increased Cbln1 levels upon AKT inhibition, further showing that in Cgcs AKT is involved in controlling presynaptic differentiation. Subsequently, *Pkn1* knockout results in enhanced axonal outgrowth and reduced presynaptic differentiation in Cgcs in vitro, both of which could be restored to WT levels upon inhibition of AKT. In line with our in vitro data, *Pkn1^–/–^* animals showed pronounced AKT phosphorylation and higher NeuroD2 levels at developmental stages critical for PF growth and synapse maturation. Throughout cerebellar development NeuroD2 is expressed only in Cgcs and ML interneurons, not in PCs (40, 41), and owing to the relatively low ML interneuron numbers compared with Cgcs, our analysis of protein extracts mainly reflects Cgc protein levels. NeuroD2 levels are particularly high during phases of axon growth in which it prevents premature presynaptic maturation, but are degraded with increasing developmental maturation in order to drive presynaptic differentiation (30). Accordingly, NeuroD2 expression is tightly controlled, since an increase in NeuroD2 decreases cell-intrinsic neuronal excitability (42). Our data offer pioneering evidence on a developmentally regulated PKN1-AKT axis that controls NeuroD2 levels and subsequently the precise balance between axonal growth and presynaptic differentiation of Cgcs. Deletion of *Pkn1* therefore interferes with the correct PF-PC synapse formation (Figures 1–5) and results in cerebellar atrophy in adult animals (Figures 6 and 7).

The truncated C-terminal fragment of PKN1 enhances NeuroD2-mediated transcription in vitro and in mammalian cells overexpressing both proteins (43); therefore we cannot exclude that despite higher NeuroD2 protein levels in *Pkn1^–/–^* cerebella, the lack of PKN1 additionally affects NeuroD2 function. However, the fact that *Pkn1^–/–^* Cgcs show a reduced density of presynaptic sites, a typical effect of NeuroD2 (30), points toward normal NeuroD2 activity.

Many neurological disorders have been linked with aberrant AKT signaling caused by germline mutations of certain tumor suppressor genes such as phosphatase and tensin homolog (*Pten*) or tuberous sclerosis proteins 1 and 2 (*Tsc2*/*Tsc1*) (44–46). Interestingly, a partial knockout of disabled homolog 2–interacting protein (*Dab2ip*), a molecule that shifts the balance of PI3K/AKT–mediated cell survival toward apoptosis signal–regulating kinase 1–mediated (ASK1-mediated) apoptosis, has a similar phenotype to *Pkn1^–/–^* mice (47). *Dab2ip*-knockdown mice show aberrant PC dendrite maturation and a defective balance of CF/PF synaptic markers. *Pkn1^–/–^* mice also show some overlapping features with mice with a deletion of *Pten* (48–50). These include increased AKT phosphorylation, abnormal axonal outgrowth, enhanced presynaptic spacing, dendritic thickening, reduction of ML thickness, degeneration of PCs, and deficits in motor coordination (48–50). However, *Pkn1^–/–^* and *Pten^–/–^* phenotypes differ in other aspects, such as brain enlargement and enlarged cell somata. The tight developmental regulation of PKN1-mediated AKT suppression (P4–P15; Supplemental Figure 5A and Figure 5) may serve as a direct explanation for this discrepancy. Despite the rapidly increasing literature on AKT signaling and neurodevelopment, this is, to our knowledge, the first report linking developmental AKT activity with NeuroD2 levels and PF-PC synapse formation, and we offer PKN1 as a regulator of this pathway. The detailed elucidation of the molecular mechanism of AKT-mediated increase in NeuroD2 protein levels, such as whether AKT enhances NeuroD2 expression via enhancement of neurogenin 1 or NeuroD1 transcriptional activity (35, 36, 51) or else protects its proteolytic degradation, remains to be solved in future investigations.

Another well-characterized effect of a defective PF-PC synapse formation is a late-onset degeneration of Cgcs and PCs (22, 37). In agreement with that, we show that the long-term effect of *Pkn1* knockout results in cerebellar shrinkage and PC degeneration and is accompanied by gait abnormalities, hind-limb clasping, and motor coordination problems (Figures 6 and 7), reminiscent of mild cerebellar ataxia (18). Interestingly, recent studies have connected microdeletions on chromosome 19p13.12, including *PKN1*, to human cerebellar hypoplasias and psychomotor delays (52–55). It has therefore been suggested that one or more of the genes on chromosome 19p13.12 have a role in the control of movements (55), and our results establish *PKN1* as a promising new candidate gene for this.

## Methods

Further information can be found in Supplemental Methods, available online with this article; https://doi.org/10.1172/JCI96165DS1

### Animals.

The generation of *Pkn1*-knockout mice (*Pkn1^–/–^* mice) has been described recently (17). Mice were backcrossed to C57BL/6N for more than 8 generations. C57BL/6N WT and C57BL/6N *Pkn1^–/–^* animals were derived from the same heterozygous crosses and then bred separately, but kept under the same housing and experimental conditions in the same room. C57BL/6N were derived from The Jackson Laboratory.

### Behavioral phenotyping.

Experiments were performed in a cohort of adult (3–9 months) WT (*n* = 11) and *Pkn1^–/–^* (*n* = 12) animals between 8 am and 1 pm.

### Hind-limb clasping.

Mice were lifted for 10–20 seconds by grasping of their tail, and movement of hind limbs was scored as previously described (56). Hind-limb clasping was assessed 3 times; the mean score was calculated and rounded up or down to the full score. In case of 0.5 or 1.5 the score was downgraded to 0 or 1.

### Ledge test.

For the ledge test, mice were lifted from their home cage and placed on another cage ledge, as described previously (56). The test was repeated twice, and the mean score of both performances was calculated and rounded up or down to the full score. In case of 0.5 or 1.5 the score was downgraded to 0 or 1.

### Pole climb.

The task for the mice in this test was to turn around and climb down from the top of a vertical pole (1 cm diameter, 60 cm height) within 120 seconds. The behavior on the pole (climbing down, sliding down sideways, freezing on the column for more than 120 seconds, or falling down) was assessed in 3 different trials, and the percentage of each behavior for each mouse was analyzed.

### Beam walk.

Motor coordination and balance were assessed by measurement of the ability of the mice to traverse a narrow 18-mm-wide, 9-mm-high, 2-m-long beam. Mice were placed in the middle, 70 cm above the ground. Illumination was set to 150 lux. The latency to traverse the beam to the safety platform was recorded for 2 trials, and the mean was calculated.

### Wire hang.

The animal was placed on a wire cage lid, which was then inverted and suspended above the home cage after a modified method (57). The cage lid was kept at 40 cm above a cage filled with soft material. The latency for each animal to release the grip with a cutoff time of 150 seconds as well as the hind-limb grip duration was recorded. The latter was performed on video recordings of 9 WT and 12 *Pkn1^–/–^* animals.

### Footprint analysis.

The entire forelimbs of mice (paw and toes) were painted in red body paint, and the entire hind limbs were painted in blue body paint. Mice were released onto a white paper cardboard into a metal tunnel (6 × 70 cm) and allowed to walk to the other end into a safe cage. For assessment of tip toe walking, we made sure that the mice could walk on the whole foot (paw and toes) and then scored the gait for how they preferred to walk (tip toes or whole foot). We calculated the toe spread score by assigning values between 0 (narrowest spread) and 2 (widest spread).

### Paraformaldehyde perfusion.

Mice were deeply anesthetized by an overdose of thiopental (150 mg/kg), and brains were fixed by transcardial perfusion with PBS (50 mM PBS, pH 7.2; 2 minutes) followed by 4% paraformaldehyde (PFA) in PBS (10 minutes).

### Preparation of cerebellar granule cells.

Cgcs were prepared as described previously (58). Cells were kept in Neurobasal medium (Thermo Fisher Scientific) supplemented with 1% penicillin/streptomycin/glutamine (Sigma-Aldrich), 2% B-27 (Thermo Fisher Scientific), and an additional 20 mM KCl. Coverslips and dishes were coated with poly-l-ornithine or poly-l-lysin (2 hours to overnight; Sigma-Aldrich), and for coverslips laminin (10 μg/ml; Sigma-Aldrich) was subsequently added for 2–3 hours at 37°C. MK-2206 (Santa Cruz Biotechnology) was added 2–3 hours after preparation.

### Transfections of cerebellar granule cells.

Nucleofection (Lonza) of Cgcs was performed as described previously (59) with 5 μg plasmid DNA and, if indicated, together with 3 μg pmax *GFP* plasmid (Lonza) using program G-013. Human *PKN1* (h*PKN1*) was purchased from GeneCopoeia and subcloned into the mammalian expression vector pEF-neo. For Lipofectamine transfections, 2 μl Lipofectamine (Thermo Fisher Scientific) and 0.9 μg plasmid DNA were mixed with Neurobasal medium and added to coverslips for 6–8 hours. The medium was then exchanged for the preconditioned culture medium, and cells were analyzed after 48 hours. *Akt123* and control siRNAs were purchased from Dharmacon. For siRNA transfections, 101 nM of each *Akt* siRNA (303 nM in total) and 303 nM control, nontargeting siRNAs were transfected with program G-013 or program O-005, both of which yielded similar results. Using higher siRNA concentrations resulted in cell death upon *Akt* knockdown (data not shown). Transfections of the same Cgc preparation with 2 different programs were counted as separate experiments.

### Cryosectioning.

P1, P4, P8, P15, and adult brains were fixed in 4% PFA for 2.5 hours (P1, P4, and P8), for 5 hours (P15), overnight (adult brains), or by PFA perfusion. Brains were incubated in a sucrose gradient (10% sucrose for 2 hours, 20% sucrose overnight, and 30% sucrose for a minimum of 2 days), embedded in OCT compound (Thermo Fisher Scientific), and stored at –80°C until analysis. Twenty-micrometer-thick sections were cut with a cryostat (CM1950, Leica), transferred onto lysine-coated coverslips (Sigma-Aldrich), and allowed to dry for 2 hours at room temperature for further analysis or stored at –20°C. In order to ensure comparable results, age-matched WT and *Pkn1^–/–^* sections were prepared on the same day and transferred onto the same coverslip.

### Immunofluorescence staining.

Cells were fixed (4% PFA 15 minutes, methanol 30 seconds at –20°C), permeabilized (0.3% Triton X-100, 15 minutes), and blocked (1% BSA, 2% goat serum, 1 hour). For cerebellar sections, permeabilization was 30 minutes and a higher blocking solution was used (10% goat serum, 2% BSA, 1 hour). For VGlut2 and p-AKT T308 staining of cerebellar sections, we performed antigen retrieval in a 10-mM sodium citrate buffer, pH 6, with 0.05% Tween-20 (100°C for 10 minutes). Primary antibodies (diluted in 0.1% Triton X-100, 1% BSA, 2% goat serum in PBS) were added at 4°C overnight. After washing in PBS, secondary antibodies (goat anti-rabbit–Alexa Fluor 488 and goat anti-mouse–Alexa Fluor 555) were added for 2–4 hours at room temperature. Coverslips/sections were washed in PBS and embedded in Mowiol (Sigma-Aldrich). Images were taken with a widefield (Axio, Zeiss) or laser scanning confocal microscope (SP5, Leica). For widefield microscopy the same exposure time and for confocal microscopy the same laser intensity were used to compare WT and *Pkn1^–/–^* sections. Confocal stacks were merged in ImageJ (NIH), and for better visualization the intensity of calbindin and VGlut2 stainings was increased to the same extent using ImageJ. For comparison of p-AKT intensity, brain sections of WT and *Pkn1^–/–^* animals were prepared on the same day, transferred onto the same coverslip, and stained with the same antibody solution. Confocal images of at least 3 independent experiments taken at the same exposure time are shown.

### Analysis of cerebellar morphology.

All analyses were performed in the cerebellar vermis area of cerebellar lobule IV/V in a blinded manner using ImageJ. The mean vermis area was calculated from 2–6 sections per animal. The IGL thickness (3–5 measurements of 2–5 sections per animal) and ML thickness (3–5 measurements of 2–5 sections per animal) were measured at the thickest part of cerebellar lobule IV/V. The VGlut2/ML ratio was determined by 4–14 measurements of 1–2 sagittal sections per animal. Perisomatic VGlut2 staining was scored by 2 experimenters in 1–2 confocal images per animal.

### Measurement of axonal length and presynaptic spacing.

Cgcs grown on laminin-coated coverslips were fixed at indicated time points and stained for TAU. Pictures were manually cleaned from background noise using ImageJ. Incomplete neurons as well as neurons with axons that crossed other axons (in that case the neuron with the shorter axon or the one that crossed more than 2 other axons was deleted) were erased. A minimum of 70 cells per coverslip (DIV1) and 20 cells per coverslip (DIV7) and a minimum of 21 GFP-positive transfected cells were analyzed with WIS-NeuroMath (60, 61). The number of presynaptic varicosities per 50-μm axonal section was determined by 1–4 measurements per axon from 24–42 cells per preparation.

### Measurement of p-AKT and NeuroD2 intensity in transfected cerebellar granule cells.

*Pkn1^–/–^* Cgcs grown on laminin were Lipofectamine-transfected with h*PKN1* on DIV5 and fixed and stained for HA and p-AKT T308 on DIV7. For NeuroD2 intensity measurements, nucleofection was used to introduce h*PKN1* in *Pkn1^–/–^* Cgcs immediately after preparation, and cells were then kept for DIV4 on laminin-coated coverslips. Cells were fixed and stained for NeuroD2 and HA. Images were taken with a widefield (Axio, Zeiss) microscope, and mean p-AKT or NeuroD2 levels were measured with ImageJ in the transfected (HA-positive) cell and the surrounding untransfected cells. Between 8 and 32 transfected cells were analyzed and averaged per experiment. The average p-AKT or NeuroD2 intensity in untransfected cells was calculated from 3–15 cells surrounding the transfected cells per picture.

### Electrophysiology.

The age for spontaneous ePSC recordings was P13–P15 (WT average age of 13.7, *Pkn1^–/–^* mice average age of 15), and for CF-induced ePSCs, P15–P17 (WT average age of 15.7, *Pkn1^–/–^* mice average age of 15.2). Animals were anesthetized (isoflurane) and decapitated. Brains were gently removed and immediately chilled (~0°C) in high-glucose artificial cerebrospinal fluid (HiGluc-aCSF) containing (in mM): NaCl, 125; NaHCO_3_, 26; d-glucose, 25; KCl, 2.5; NaH_2_PO_4_, 1.43; CaCl_2_, 2; MgCl_2_, 1. The aCSF pH was adjusted to 7.4 with a saturating carbogen mix (95 O_2_/5% CO_2_). Parasagittal cerebellar slices 300 μm thick were cut along the vermis with a vibratome (Leica VT1200S) in ice-cold HiGluc-aCSF. Slices were thereafter allowed to recover for at least 30 minutes in HiGluc-aCSF and bubbled with 95% O_2_/5% CO_2_ at room temperature until used and were kept for a maximum of 8 hours. Whole-cell patch recordings were performed on visually identified PCs in lobulus IV/V using a ×20 water immersion objective (Olympus) with an additional ×2 magnifier as described previously (62). Briefly, slices were transferred to a submersion-style recording chamber mounted on an Olympus BXS1WI and superfused with standard aCSF, continuously bubbled with 95% O_2_/5% CO_2_ at room temperature, comprising (in mM): NaCl, 125; NaHCO_3_, 26; d-glucose, 10; KCl, 2.5; NaH_2_PO_4_, 1.43; CaCl_2_, 2; MgCl_2_, 1. The aCSF had an osmolality of 310 mOsm/kg. Patch pipettes were pulled from borosilicate capillaries (GB120F-10, Science Products) with a Sutter P-1000 puller; the resistance was typically 2–5 MΩ when filled with internal solution consisting of (in mM): K-gluconate, 132; EGTA/KOH, 1; MgCl_2_, 2; NaCl, 2; HEPES/KOH, 10; Mg-ATP, 2; GTP, 0.5. The pH was adjusted to 7.2–7.25 with KOH, and the osmolality was 280–285 mOsm/kg. Recordings were obtained using a Multiclamp 700B amplifier and displayed with pClamp (Molecular Devices). In whole-cell configuration (holding potential –70 mV), series resistances were typically around 10–15 MΩ. Cells were allowed to equilibrate for 10 minutes before recording. Evoked CF inputs were triggered with a stimulation electrode filled with aCSF (resistance ~0.5 MΩ) that was placed 50–70 μm away from the recorded PC soma in the granule cell layer. The unipolar square pulses with durations of 0.2 milliseconds were delivered at 0.1 Hz via a constant current stimulus isolator (World Precision Instruments A365). Stimulation current amplitudes were between 5 μA and 100 μA. At least 3 discrete steps above triggering threshold with a step size of 10 μA were recorded in each cell. The CF synaptic inputs to the PCs were recorded at room temperature at a holding potential of –70 mV, low-pass-filtered at 10 kHz, and sampled at 20 kHz. Data analysis was done in Matlab (Mathworks), and traces were analyzed by 2 different blinded experimenters. Spontaneous ePSCs and iPSCs were recorded at room temperature, low-pass-filtered at 10 kHz, and sampled at 20 kHz; traces were low-pass-filtered after recordings at 1–4 kHz and analyzed with Clampfit module in pClamp.

### Western blotting.

Protein extracts and Western blotting were performed as described previously (59). Primary antibodies were added overnight in 5% BSA in TBS-T at 4°C, and secondary antibodies (LI-COR) or HRP-tagged antibodies were added for 90 minutes in 5% milk in TBS-T. After washing in TBS-T, membranes were imaged and analyzed with Odyssey Infrared Imager (LI-COR) or ECL detection as described previously (63).

### Antibodies.

Clone numbers, where known, and catalog numbers are provided in parentheses. The p-AKT T308 antibody (catalog SAB4300043, further validated with MK-2206 in Cgcs; not shown) was from Sigma-Aldrich. The following antibodies were from Cell Signaling: ERK1/2 (catalog 9102), GAPDH (D16H11, catalog 5174), HA-Tag (C29F4, catalog 3724), NeuN (D4G40, catalog 24307), p-AKT S473 (Western blotting, D9E, catalog 4060), p-ERK1/2 (catalog 9101), pan-AKT (Western blotting, 40D4, catalog 2920), synapsin-I (D12G5, catalog 5297), and TAU (Tau46, catalog 4019). α-Tubulin (DM1A, catalog ab80779), calbindin (catalog ab11426), calbindin (CB-955, catalog ab82812), cerebellin 1 (EPR13649, catalog ab181379), Ki-67 (catalog ab15580), p-AKT S473 (immunofluorescence staining, EP2109Y, catalog ab81283, further validated with MK-2206 in Cgcs; not shown), AKT1/2/3 (immunofluorescence staining and Western blotting, EPR16798, catalog ab179463), and VGlut2 (8G9.2, catalog ab79157) were from Abcam. HA.11 (clone 16B12, catalog MMS-101P) was from Covance. VGlut1 (catalog 48-2400), goat anti-rabbit–Alexa Fluor 488 (catalog A11070), and goat anti-mouse–Alexa Fluor 555 (catalog A21425) were purchased from Thermo Fisher Scientific, and GluD2 (D-9, catalog sc-393437) and NeuroD2 (G-10, catalog sc-365896) were from Santa Cruz Biotechnology. PKN1 (clone 49/PRK1, catalog 610687) was from BD Transduction Laboratories. The secondary antibodies for the Odyssey Infrared Imager, IRDye 680RD (catalog 926-68072) and IRDye 800CW (catalog 926-32211), were obtained from LI-COR.

### Statistics.

No statistical methods were used to predetermine sample sizes, but our sample sizes are similar to those reported in previous publications and typically had a statistical power sufficient to detect differences on the order of our effect sizes. Normal distributions of data were presumed but not formally tested; equal variances were tested, and if they were not met, an unpaired 2-tailed *t* test with Welch’s correction was used. All data are presented as individual *n* values with or as mean ± SEM. For behavioral and histochemical analysis, *n* values refer to different animals from at least 3 different litters. For all cell culture experiments, *n* values refer to animals from different litters or transfections. For comparison of 2 independent groups, a 2-tailed unpaired *t* test was used, and for comparison of 2 dependent groups, a 2-tailed paired *t* test was used. For comparison of 3 or more groups, a 1-way ANOVA with Newman-Keuls multiple-comparisons test was used, and for comparison of 2 variables of 2 groups, a 2-way ANOVA was used. For comparison of noncontinous data (i.e., behavior, CF innervation), a χ^2^ test was used. *P* values smaller than 0.05 were considered as statistically significant. All analyses were performed in GraphPad Prism 5 and 7.

### Study approval.

For all studies using adult animals, male mice were used, and all procedures were approved by the Austrian Animal Experimentation Ethics Board in compliance with the European Convention for the Protection of Vertebrate Animals Used for Experimental and Other Scientific Purposes (ETS no. 123) (BMWFW-66.011/0040-WF/V/3b/2016). Every effort was made to minimize the number of animals used. The study was designed in compliance with Animal Research: Reporting of In Vivo Experiments (ARRIVE) guidelines. Blinding was always performed by a third party, and every figure legend clearly states whether analysis was performed in a blinded or nonblinded manner.

## Author contributions

GBB and SzN developed the study concept and design. SzN performed immunoblotting, immunohistochemistry, and cell and slice culture experiments. RE and SzN performed studies on cerebellar morphology. FF and GB performed recombinant DNA work. AJMC and PJP contributed the *Pkn1^–/–^* mice. CS designed and SzN, CS, RE, and GBB performed the animal behavior experiments. LZ, HS, and AK performed the electrophysiological experiments. SzN and GBB supervised the project and wrote the manuscript with critical input from all authors. GBB was the responsible coordinator of the project. All authors approved the manuscript.

## Supplementary Material

Supplemental data

## Version 1 03/01/2018

In-Press Preview

## Version 2 04/16/2018

Electronic publication

## Version 3 05/01/2018

Print issue publication
