# Supplementary material for: Protein kinase N1 critically regulates cerebellar development and long-term function
Source: J Clin Invest. 2018 Apr 16;128(5):2076–88. doi: 10.1172/JCI96165 (PMC5919825; doi:10.1172/JCI96165)
Supplement: Supplemental data [file jci-128-96165-s001.pdf]

SUPPLEMENTAL DATA

Supplemental Methods:

Preparation of organotypic cerebellar slices

Organotypic slices were prepared from P11-P12 WT and *Pkn1*$^{-/-}$ animals as described previously (1). 350 µm thick sagittal cerebellar slices were cut with a McIllwain tissue chopper (Tedpella), transferred into ice-cold Gey-solution (Sigma, supplemented with 0.45% Glucose, 1% Penicillin/Streptomycin/Glutamine) and if necessary manually separated with a fine scalpel. Slices were washed twice in culture medium (MEM, 25% Horse serum, 2.5% HEPES, 1% Penicillin/Streptomycin/Glutamine) and transferred onto culture inserts (Merck Millipore, cell culture insert, hydrophilic PTFE). 5 µM MK-2206 was added 2-3 h after preparation. Medium was exchanged every 2-3 days and slices were kept for 6-9 days in vitro (DIV).

Supplemental Figure Legends:

Supplemental Figure 1. (**A**) Western bot analysis of whole cerebellum protein extracts revealed that the ratio of VGlut2 to VGlut1 dropped in WT animals from P8 to P15, but not in *Pkn1*$^{-/-}$ animals (one-way ANOVA with Newman-Keuls multiple comparison test, $F(3,13) = 4.211$, $P = 0.0275$, post-test (*) $P < 0.05$, n = 4-5 WT/4-5 *Pkn1*$^{-/-}$ whole cerebella protein extracts from 4-5 litters/group). Compared to WT VGlut1 expression levels were consistently lower throughout development in *Pkn1*$^{-/-}$ animals, but this did not reach statistical significance (two-way ANOVA analysis, genotype $F(1,36) = 2.57$, $P = 0.1178$, age $F(4,36) = 15.14$, $P < 0.0001$, interaction $F(4,36)$ $= 0.11$, $P = 0.9784$, n = 4-5 WT/4-5 *Pkn1*$^{-/-}$ whole cerebella protein extracts from > 3 different litters/group). Full unedited Blot is shown. See Figure 6F for representative western blots of adult

animals. Data is presented as mean±SEM. (**B**) Thickness of Purkinje cell dendrites of P8 and P15 old animals was measured at a distance of 50-70 µm from the soma (the mean of 31-142 measurements of 2-7 sagittal sections/animal is shown, one-way ANOVA with Newman-Keuls multiple comparison test, $F(3,20) = 20.82$, $P < 0.0001$, post-test (***) $P < 0.001$, $n = 5$ WT/5 *Pkn1*$^{-/-}$ animals for P8 and $n = 6$ WT/8 *Pkn1*$^{-/-}$ animals for P15 from 5-6 litters/group). Data is presented as individual *n*-values with mean±SEM. (**C**) The thickness of the external granule layer (EGL), molecular layer (ML), internal granule layer (IGL) or sagittal area of the vermis is not different between WT and *Pkn1*$^{-/-}$ animals during development (P1-P15). Representative cresyl violet-stained sections are shown left to the table. Data in the table is presented as mean±SEM. *n*-values are given in brackets and are derived from 3 or more different litters/group. Analysis was performed on 2-6 sagittal sections/animal. An unpaired t-test was used for analysis of statistical significance ($P > 0.05$ for all comparisons). (**D**) Spontaneous excitatory (e) PSCs were recorded from Purkinje cells in cerebellar slices prepared from P13-P15 old animals. ePSC amplitudes were determined (two-tailed unpaired t-test, $t(8) = 1.088$, $P = 0.384$, $n = 6$ WT/4 *Pkn1*$^{-/-}$ cells from 3-5 animals/group). Data is presented as individual *n*-values with mean±SEM. (**E**) Table summarizing inhibitory (i) postsynaptic currents (PSCs) recorded from whole-cell patch clamp recordings of P13-P15 WT and *Pkn1*$^{-/-}$ PC. Data for frequencies and amplitudes is presented as mean±SEM for WT cells or as individual values for *Pkn1*$^{-/-}$ cells. See Figure 1E for example traces. Analysis in **B**, **C, D** and **E** was done by an experimenter blinded to the genotype.

Supplemental Figure 2. (**A**) In *Pkn1*$^{-/-}$ Cgc cotransfected with human HA-tagged *PKN1* (h*PKN1*) and *GFP*, HA-staining was found along en passant swellings of the axon. Image is representative of 3 separate experiments. (**B**) HA-staining was further found in the cytosol and dendrites. Image

is representative of 3 separate experiments. (**C**) Cgc protein extracts were probed for phospho-AKT (pAKT) [S473] (two-way ANOVA analysis, genotype $F(1,43) = 11.84$, (**) $P = 0.0013$; time $F(3,43) = 4.63$, (**) $P = 0.0068$; interaction $F(3,43) = 1.46$, $P = 0.240$, $n = 7$ WT/6 *Pkn1*$^{-/-}$ Cgc preparations from 6-7 litters/group) and pAKT [T308] (two-way ANOVA analysis, genotype $F(1,40) = 4.57$, (*) $P = 0.0387$; time $F(3,40) = 9.17$, (***) $P < 0.0001$; interaction F(3,40) = 0.65, $P = 0.5865$, $n = 7$ WT/5 *Pkn1*$^{-/-}$ Cgc preparations from 5-7 litters/group). Data is presented as mean±SEM. (**D**) pAKT [S473] intensity was measured in untransfected *Pkn1*$^{-/-}$ Cgc and *Pkn1*$^{-/-}$ Cgc expressing human HA-tagged PKN1 (hPKN1) at DIV7 (5-8 transfected cells were analyzed/experiment, two-tailed paired t-test, $t(2) = 6.045$, (*) $P = 0.0263$, $n = 3$ from 3 litters). Data is presented as individual *n*-values. (**E**) pAKT [T308] intensity was measured in untransfected *Pkn1*$^{-/-}$ Cgc and *Pkn1*$^{-/-}$ Cgc expressing GFP at DIV7 (16-19 transfected cells were analyzed/experiment, two-tailed paired t-test, $t(2) = 0.1298$, $P = 0.9086$, $n = 3$ from 3 litters). Data is presented as individual *n*-values. Experimenters were not blinded to the genotype.

Supplemental Figure 3. (**A**) 24 h treatment of Cgc with MK-2206 dose-dependently reduced the intensity of pAKT [T308] in western blot analysis at DIV1 (pAKT [T308]/AKT for Control and 1µM MK-2206: $n = 3$ WT/3 *Pkn1*$^{-/-}$ Cgc from 3 litters/group; 5µM MK-2206 $n = 2$ WT/2 *Pkn1*$^{-/-}$ Cgc from 2 litters/group, two-way ANOVA analysis, source of variation: genotype $F(1,10) = 0.33$, $P = 0.5782$; MK-concentration $F(2,10) = 11.74$, (**) $P = 0.0024$, interaction $F(2,10) = 0.26$, $P = 0.7739$). For downregulation of pAKT [S473] by MK-2206 see Figure 3E and Supplemental Figure 3C. Phospho-(p) ERK refers to extracellular signal regulated kinase. Full unedited Blot is shown. Data is presented as mean (where $n = 2$) or mean±SEM. (**B**) NeuroD2 intensity was measured in untransfected *Pkn1*$^{-/-}$ Cgc and *Pkn1*$^{-/-}$ Cgc expressing GFP at DIV4 (43-50 transfected cells were

analyzed/experiment, two-tailed paired t-test, $t(2) = 0.2479$, $P = 0.8273$, $n = 3$ from 3 litters). Data is presented as individual n-values. (**C**) 24 h treatment of Cgc with MK-2206 dose-dependently increased Cbln1 expression in WT animals at DIV1 (one-way ANOVA with Newman-Keuls multiple comparison test, $F(2,8) = 20.58$, $P = 0.0007$, post-test (*) $P < 0.05$, (**) $P < 0.01$, (***) $P < 0.001$, $n = 3$-4 from 3-4 litters). Data is presented as individual n-values with mean±SEM. (**D**) 24 h treatment of $Pkn1^{-/-}$ Cgc with MK-2206 inhibited NeuroD2 and increased Cbln1 expression at DIV1 similar to WT animals (Blot is representative of 2-3 separate experiments). (**E**) Cgc protein extracts of WT and $Pkn1^{-/-}$ Cgc were probed for Cbln1 expression during DIV1-3 (two-way ANOVA analysis, genotype $F(1,25) = 3.75$, $P = 0.0643$; time $F(2,25) = 0.73$, $P = 0.4937$; interaction $F(2,25) = 0.78$, $P = 0.4678$, $n = 4$-7 WT/4-5 $Pkn1^{-/-}$ Cgc preparations from 4-7 litters/group). Representative blots are shown for DIV 1, 2 and 7. Data is presented as mean±SEM. Experimenters were not blinded to the genotype.

Supplemental Figure 4: (**A**) WT Cgc were transfected with siRNAs targeting *Akt123* or control non-targeting siRNAs. *Akt123* siRNAs significantly reduced pan-AKT expression at DIV1 (for representative picture see Figure 4A, 48-50 cells from 10 different pictures were analyzed/experiment, two-tailed unpaired t-test, $t(8) = 4.297$, (**) $P = 0.0026$, $n = 5$ from 3 different litters) and DIV4 (48-50 cells from 10 different pictures were analyzed/experiment, two-tailed unpaired t-test, $t(8) = 2.678$, (*) $P = 0.0280$, $n = 5$ from 3 different litters). (**B**) Similarly, $Pkn1^{-/-}$ Cgc were transfected with siRNAs targeting *Akt123* or control non-targeting siRNAs. *Akt123* siRNAs significantly reduced pan-AKT staining at DIV1 (48-50 cells were analyzed/experiment, two-tailed unpaired t-test, $t(8) = 2.451$, (*) $P = 0.0399$, $n = 5$ from 3 different litters) and DIV4 (for representative picture see Figure 4C, 48-50 cells were analyzed/experiment, two-tailed unpaired t-

test, $t(8) = 3.046$, (*) $P = 0.0159$, $n = 5$ from 5 different litters). (**C**) *Akt123* siRNAs significantly increased Cbln1 expression levels in WT Cgc at DIV1 (two-tailed unpaired t-test, $t(6) = 3.893$, (**) $P = 0.0081$, $n = 5$ from 3 different litters). (**D**) *Akt123* siRNAs significantly increased Cbln1 expression levels in *Pkn1*$^{-/-}$ Cgc at DIV1 (two-tailed unpaired t-test, $t(6) = 2.523$, (*) $P = 0.0451$, $n = 5$ from 5 different litters). All data is presented as individual *n*-values with mean±SEM. Experimenters were not blinded to the genotype or treatment.

Supplemental Figure 5. (**A**) Whole cerebellum protein extracts prepared from postnatal day (P)1-P15 old WT (see Figure 5A) and *Pkn1*$^{-/-}$ animals revealed higher (**A**$_{i}$) pAKT [T308] (two-way ANOVA analysis, source of variation: genotype $F(1,28) = 6.67$, (*) $P = 0.0153$; time $F(3,28) = 4.44$, (*) $P = 0.0113$, interaction $F(3,28) = 0.53$, $P = 0.6847$, n = 3-4 WT/4-6 *Pkn1*$^{-/-}$ whole cerebellum protein extracts from 3-6 litters/group) and (**A**$_{ii}$) pAKT [S473] in *Pkn1*$^{-/-}$ animals (two-way ANOVA analysis, source of variation: genotype $F(1,28) = 4.45$, (*) $P = 0.0440$; time $F(3,28) = 4.40$, (*) $P = 0.0118$, interaction $F(3,28) = 0.53$, $P = 0.6641$, n = 3-4 WT/4-6 *Pkn1*$^{-/-}$ whole cerebellum protein extracts from 3-6 litters/group). The (**A**$_{iii}$) PKN1/GAPDH ratio in WT animals dropped from P1-P8 to P15 (one-way ANOVA with Newman-Keuls multiple comparison test, $F(3,14) = 7.688$, $P = 0.0028$, post-test (**) $P < 0.01$, n = 4-5 WT whole cerebellum protein extracts from 4-5 litters). Data is presented as mean±SEM. (**B**) There was no difference in pAKT [S473] or NeuroD2 levels in protein extracts prepared from adult (6-9 month old) WT and *Pkn1*$^{-/-}$ cerebella (n = 3 WT/3 *Pkn1*$^{-/-}$ animals from 3 different litters/group). (**C**) Cerebellar sections from adult (3-9 month old) animals stained for pAKT [S473] did not reveal any differences between WT and *Pkn1*$^{-/-}$ animals. Pictures are representative of 3 different experiments. (**D**) Organotypic slices were prepared from P11-P12 old animals and cultured for 6-9 days in vitro with or without the AKT-

inhibitor MK-2206 [5 μM]. Representative images of Calbindin-stained Purkinje cells and the distal dendritic branchlets are shown (n = 3-6, see Di and Dii for analysis). (**Di**) The thickness of Purkinje cell dendrites was measured at a distance of 50-70 μm from the soma (the mean of 1-6 measurements of 5-44 Purkinje cells from 2-6 slices/animal is shown, one-way ANOVA with Newman-Keuls multiple comparison test, $F(3,13) = 37.65$, $P < 0.0001$, post-test (**) $P < 0.01$, (***) $P < 0.001$, n = 3-4 WT/4-6 *Pkn1*$^{-/-}$ animals from 3-6 litters/group except WT MK-2206 which was derived from 3 animals of 2 litters). (**Dii**) The thickness of distal dendritic branchlets was analyzed (the mean of 2-10 measurements of 13-44 Purkinje cells from 2-6 slices/animal is shown, one-way ANOVA with Newman-Keuls multiple comparison test, $F(3,13) = 25.52$, $P < 0.0001$, post-test (***) $P < 0.001$, n = 3-4 WT/4-6 *Pkn1*$^{-/-}$ animals from 3-6 litters/group except WT MK-2206 which was derived from 3 animals of 2 litters). Data is presented as individual n-values with mean±SEM. Experimenters were not blinded to the genotype, except in (B).

Supplemental Figure 6. (**A**) There was no difference in the thickness of the proliferative (Ki-67 positive) cells in the EGL at the peak of proliferation (P8) (the mean of 10 measurements of 2 sagittal sections/animal is shown, two-tailed unpaired t-test, $t(8) = 0.08272$, $P = 0.9361$, n = 5 WT/5 *Pkn1*$^{-/-}$ animals from 3 or more litters/group). The thickness of the Ki-67 positive layer was set in relation to the thickness of the total EGL, as analyzed in Hoechst-stained sections. Data is presented as individual n-values with mean±SEM. (**B**) There were no ectopic NeuN-positive cells in the ML of adult *Pkn1*$^{-/-}$ animals (the mean of 5-15 measurements from 1 section/animal are shown, two-tailed unpaired t-test, $t(8) = 1.151$, $P = 0.2829$, n = 5 WT/5 *Pkn1*$^{-/-}$ animals from 3 or more litters/group). Data is presented as individual n-values with mean±SEM. (**C**) The number of VGlut2-stained climbing fiber varicosities in a 20x20μm square decreased from the proximal

(squares were placed adjacent to PC cell bodies) to the distal part of PC dendrites (squares were placed 10-30 μm away from the end of the ML) in adult WT animals (the mean of 3-14 measurements of 1-2 sagittal sections/animal is shown, two-tailed paired t-test, $t(11) = 2.798$, (*) $P = 0.0173$, $n = 12$ from 8 litters). This was not seen in adult *Pkn1*$^{-/-}$ animals (the mean of 6-14 measurements of 2 sagittal sections/animal is shown, two-tailed paired t-test, $t(10)=1.759$, $P = 0.1092$, $n = 11$ from 7 litters). Data is presented as individual *n*-values. (**D**) The thickness of Purkinje cells dendrites in sections prepared from adult animals was measured at a distance of 50-70 μm from the cell soma (the mean of 6-180 measurements of 2-11 sagittal sections/animal is shown, two-tailed unpaired t-test, $t(15) = 2.919$, (*) $P = 0.0106$, $n = 10$ WT/7 *Pkn1*$^{-/-}$ animals from 5-6 litters/group). Data is presented as individual *n*-values with mean±SEM. All analyses were performed by experimenters blinded to the genotype.

Supplemental Figure 7. (**A**) Open-field behavior was tested over 10 min in a 50×50 cm open field box. Illumination was set to 180 lux. The total distance travelled was analyzed using Video-Mot 2 equipment and software (TSE-systems, Bad Homburg, Germany). Experiments were performed with adult (4-9 months) mice (two-tailed unpaired t-test $t(21) = 1.341$, $P = 0.194$, $n = 11$ WT/12 *Pkn1*$^{-/-}$ animals from 4-6 litters/group). (**B**) Anxiety-related behavior was tested in the elevated plus maze over a period of 5 min. Illumination was set to 180 lux. Movement in the open, closed and neutral area was analyzed using Video-Mot 2 equipment and software in adult mice (two-way ANOVA, source of variation: genotype $F(1,63) = 0.00$, $P = 0.99930$, area $F(2,63) = 509.03$, $P < 0.0001$, interaction $F(2,63) = 0.68$, $P = 0.5087$, $n = 11$ WT/12 *Pkn1*$^{-/-}$ animals from 4-6 litters/group). All data is presented as individual *n*-values with mean±SEM. Experimenters were not blinded to the genotype.

Supplemental References:

1. Konishi Y, Stegmuller J, Matsuda T, Bonni S, and Bonni A. Cdh1-APC controls axonal growth and patterning in the mammalian brain. *Science.* 2004;303(5660):1026-30.

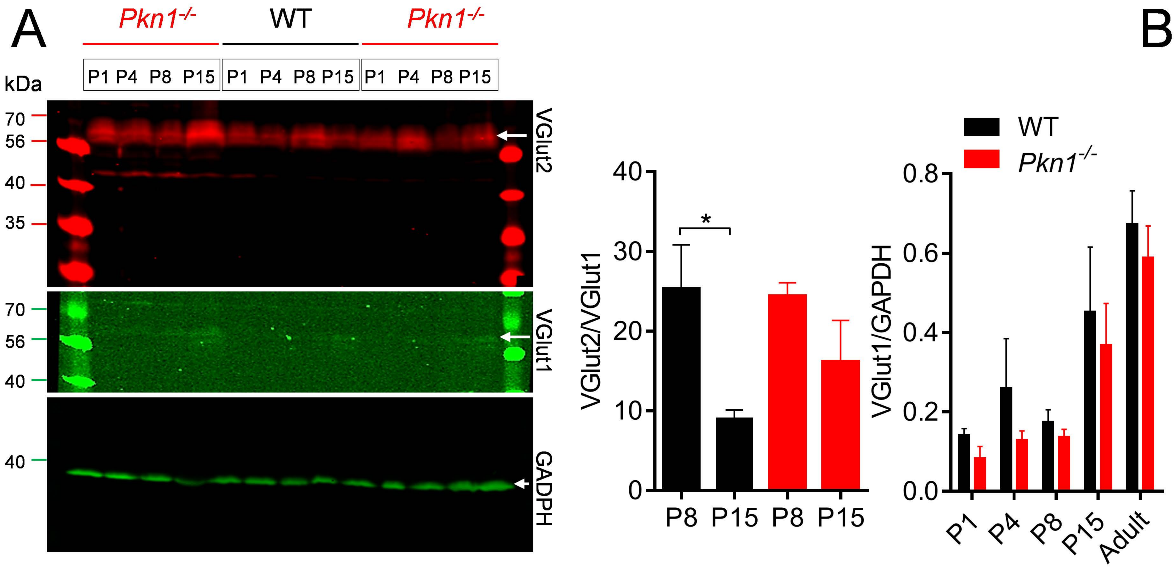

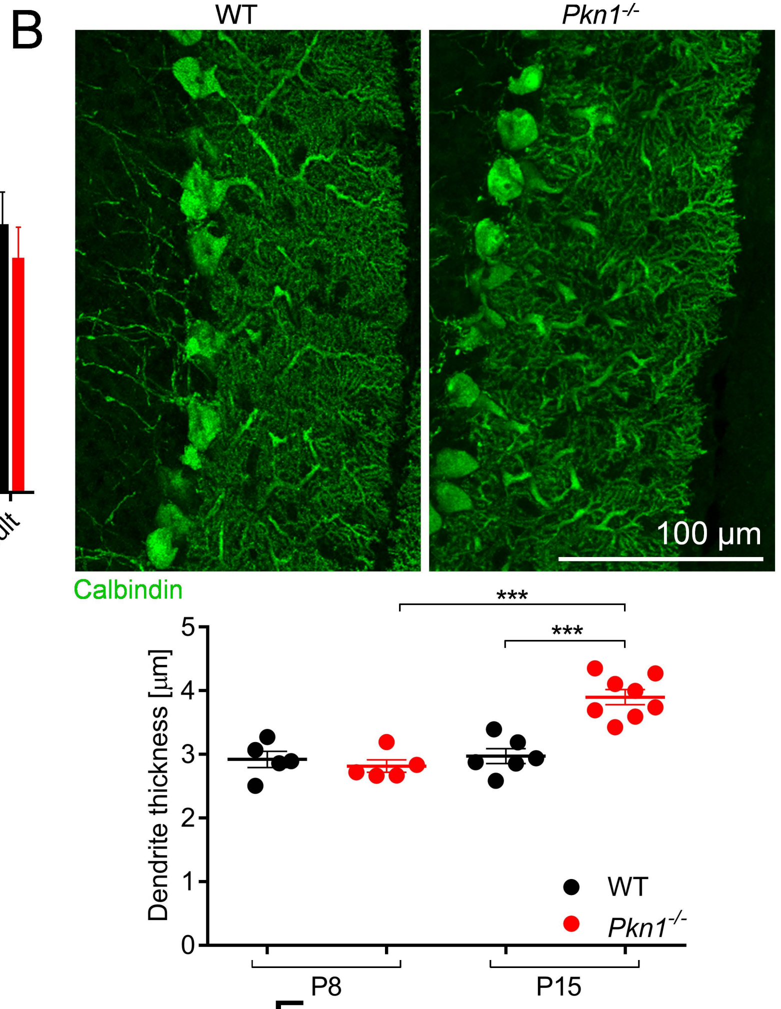

C

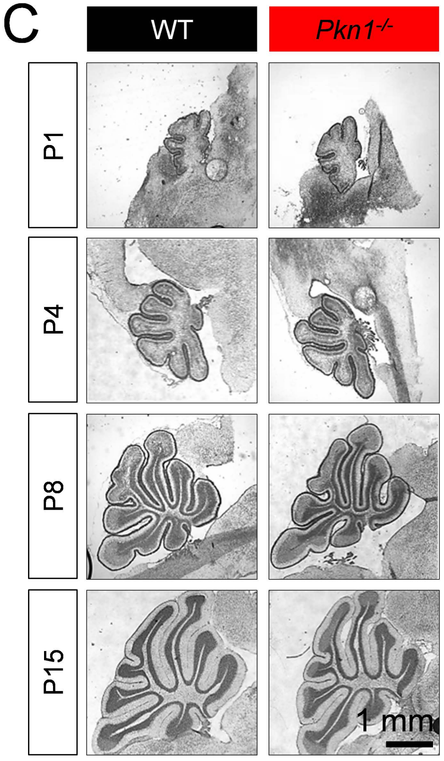

Age	Genotype	Lobule (n)	EGL [µm]	ML [µm]	IGL [µm]	Area $[mm^2]$
P1	WT	V (3)	30.23 ± 6.29	23.84 ± 2.06	33.71 ± 1.29	1.0 ± 0.05
		IX (3)	26.48 ± 7.35	19.10 ± 4.03	30.90 ± 1.10	
	Pkn1-/-	V (3)	22.59 ± 0.67	19.60 ± 2.94	28.07 ± 1.64	1.0 ± 0.05
		IX (3)	29.37 ± 1.07	22.79 ± 3.46	31.50 ± 1.80	
P4	WT	V (3)	46.59 ± 1.69	23.56 ± 2.91	38.76 ± 2.54	2.0 ± 0.1
		IX (3)	39.52 ± 1.78	28.96 ± 7.00	41.49 ± 1.28	
	Pkn1-/-	V (4)	42.76 ± 1.02	27.84 ± 1.72	35.86 ± 6.51	2.0 ± 0.1
		IX (4)	39.12 ± 2.64	21.36 ± 0.85	34.69 ± 8.18	
P8	WT	V (7)	39.63 ± 2.87	43.79 ± 2.37	138.37 ± 4.47	3.6 ± 0.1
		IX (7)	36.87 ± 2.53	58.37 ± 4.67	62.73 ± 4.51	
	Pkn1-/-	V (8)	38.45 ± 3.35	47.11 ± 1.64	127.73 ± 7.37	3.7 ± 0.1
		IX (7)	34.93 ± 2.27	48.54 ± 3.96	61.16 ± 2.74	
P15	WT	V (6)	7.76 ± 0.21	116.57 ± 5.52	144.34 ± 4.61	6.7 ± 0.2
		IX (3)	9.86 ± 1.12	115.82 ± 4.79	64.79 ± 2.19	
	Pkn1-/-	V (3)	7.60 ± 0.88	125.05 ± 9.18	143.73 ± 6.72	7.0 ± 0.3
		IX (4)	7.18 ± 1.07	109.20 ± 7.21	75.13 ± 7.64	

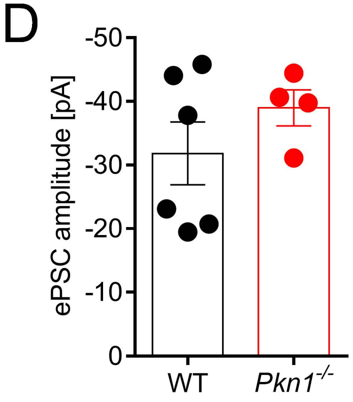

E

iPSC		
SCORE	WT	*Pkn1-/-*
YES	5	1
NO	2	3
Frequency [Hz]	1.8 ± 0.5	0.007
Amplitude [pA]	30.3 ± 0.9	36.8

Supplemental Figure 1

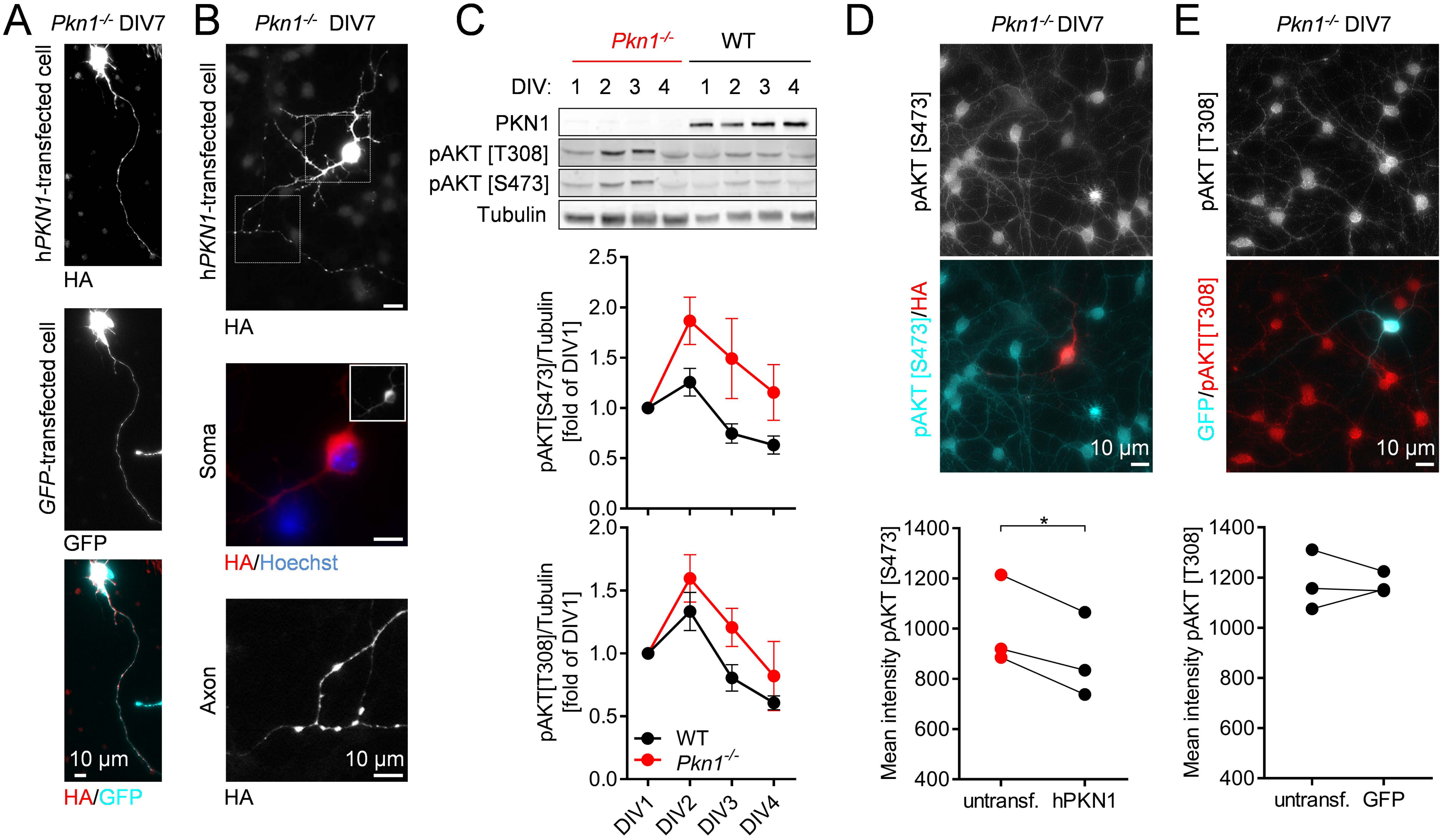

Supplemental Figure 2

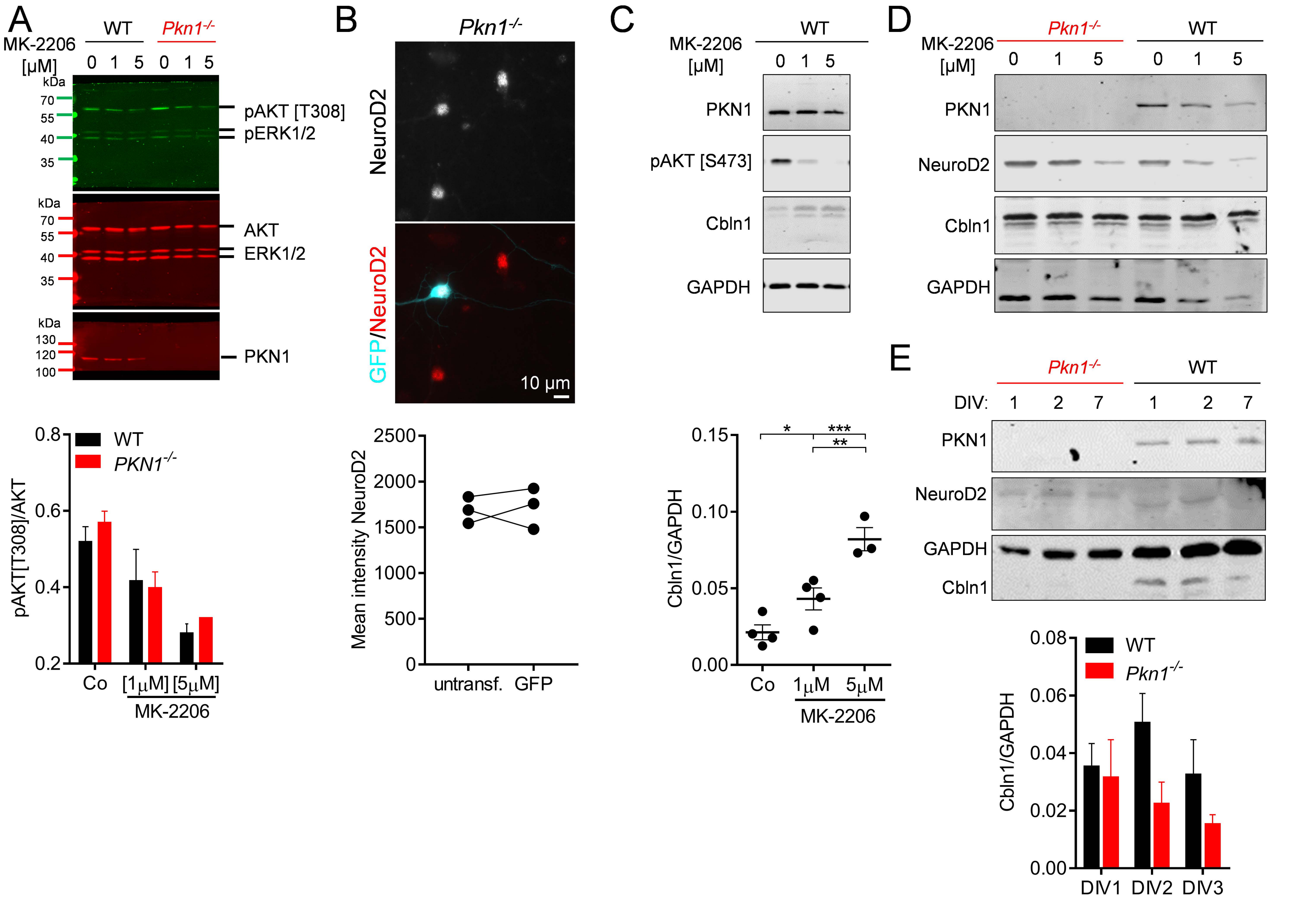

Supplemental Figure 3

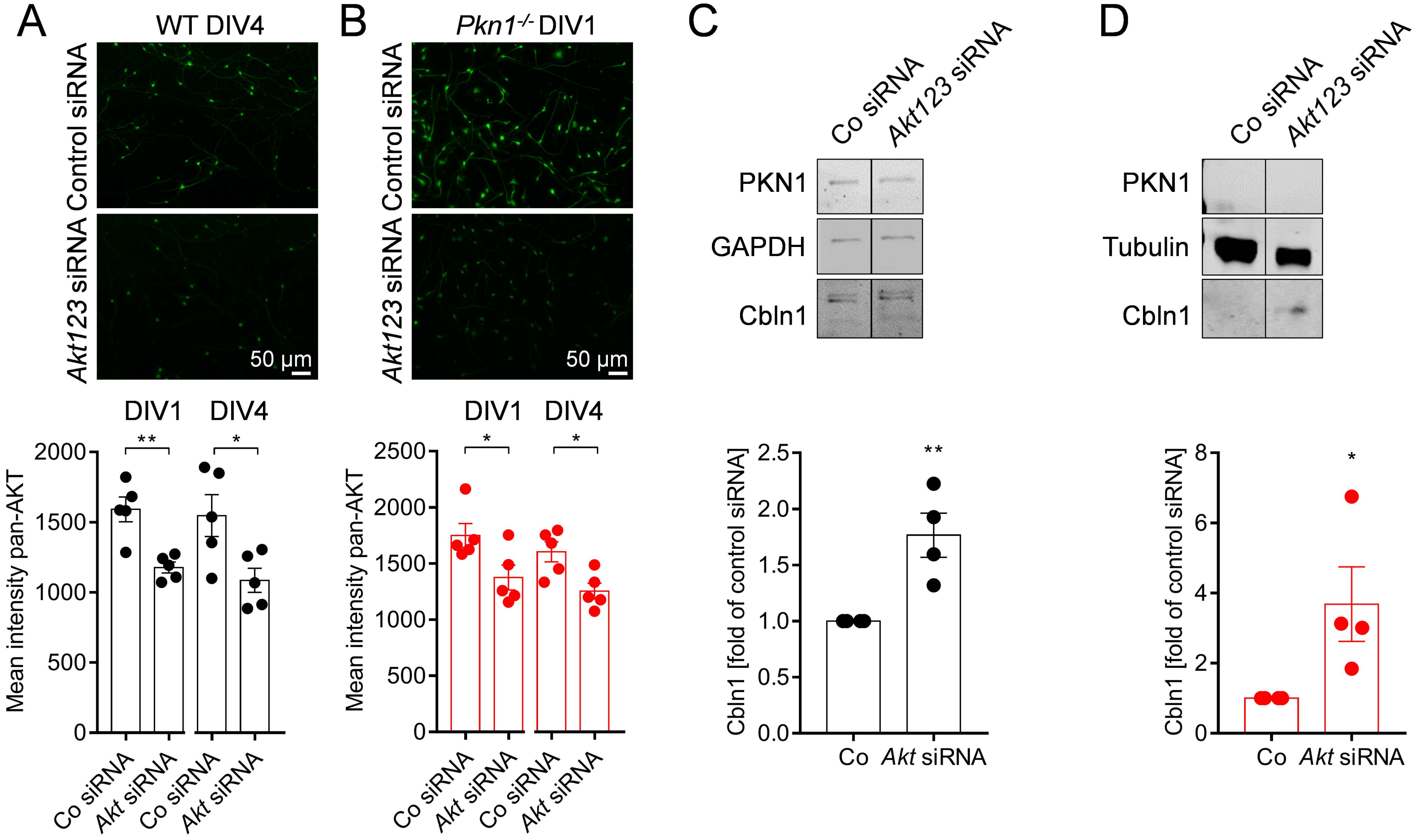

Supplemental Figure 4

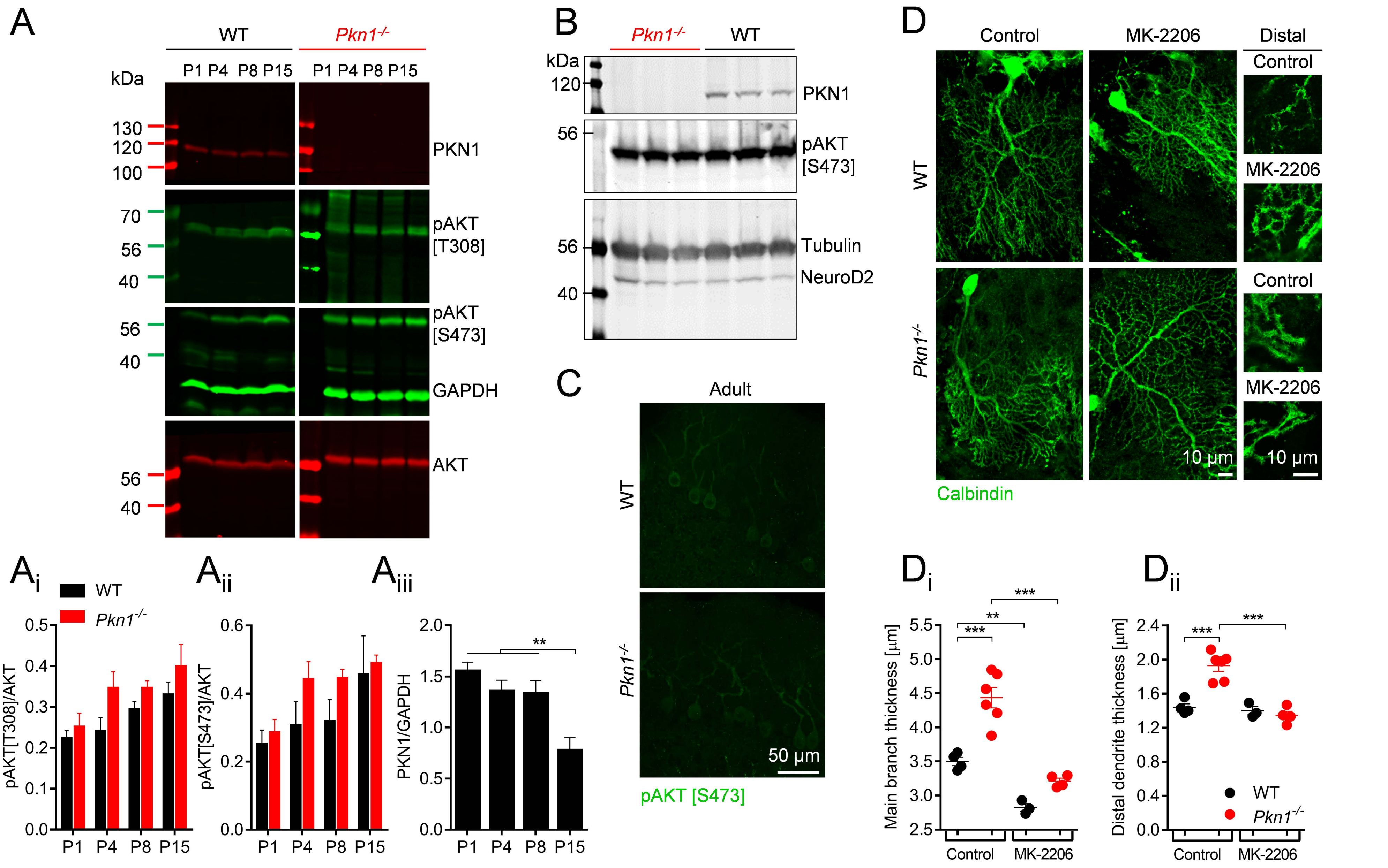

Supplemental Figure 5

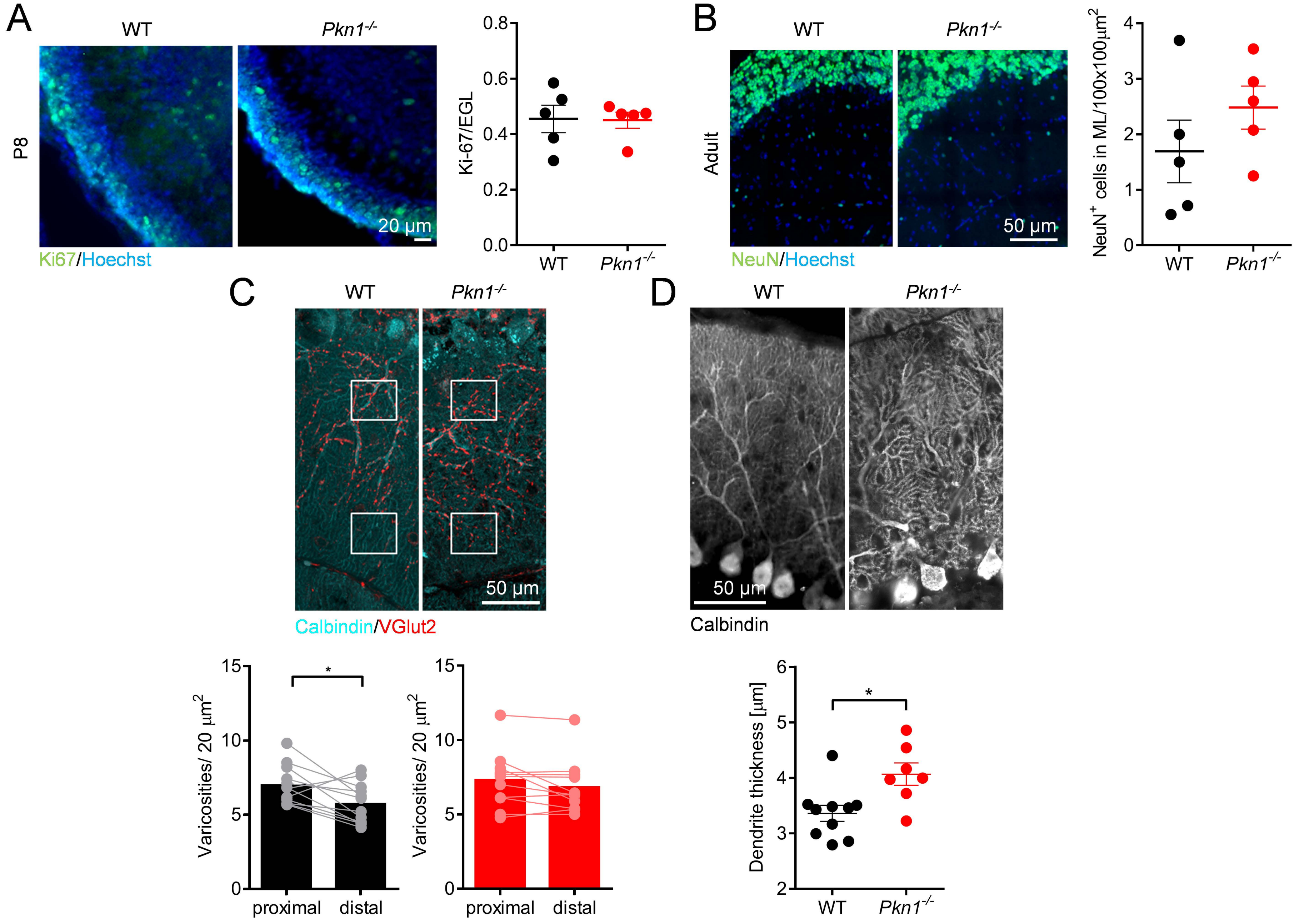

Supplemental Figure 6

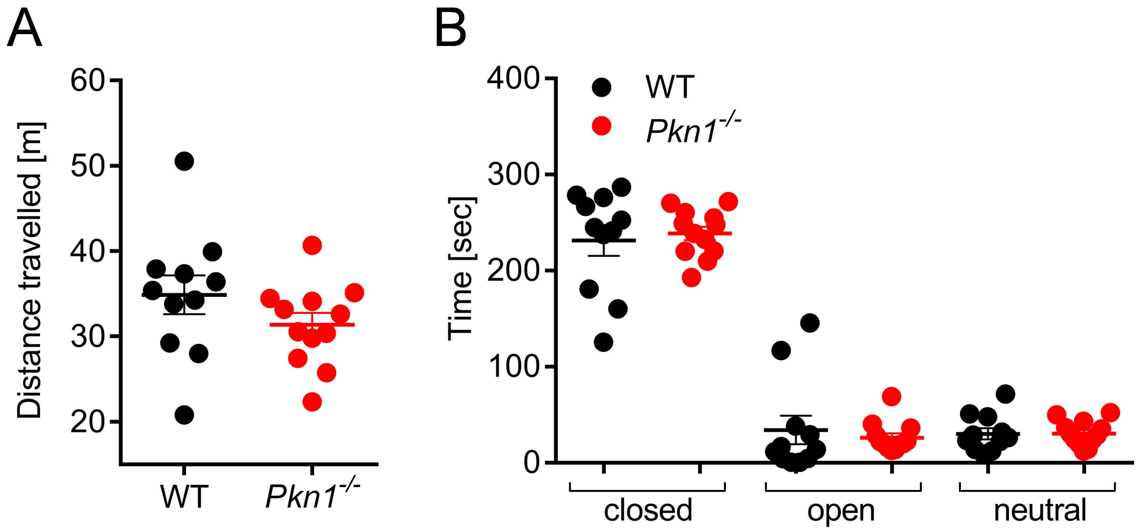

Supplemental Figure 7
